# Exposure to continuous or fluid theories of sexual orientation leads some heterosexuals to embrace less-exclusive heterosexual orientations

**DOI:** 10.1038/s41598-021-94479-9

**Published:** 2021-08-16

**Authors:** James S. Morandini, Liam Dacosta, Ilan Dar-Nimrod

**Affiliations:** 1 The School of Psychology, University of Sydney, Sydney, NSW 2006 Australia; 2 The Charles Perkins Centre, University of Sydney, Sydney, NSW 2006 Australia

**Keywords:** Psychology, Human behaviour

## Abstract

We examined whether heterosexual individuals’ self-reported sexual orientation could be influenced experimentally by manipulating their knowledge of the nature of sexual orientation. In Study 1 (180 university students, 66% female) participants read summaries describing evidence for sexual orientation existing on a continuum versus discrete categories or a control manipulation, and in Study 2 (460 participants in a nationally representative Qualtrics panel, 50% female) additionally read summaries describing sexual orientation as fluid versus stable across the life-course. After reading summaries, participants answered various questions about their sexual orientation. In Study 1, political moderates and progressives (but not conservatives) who read the continuous manipulation subsequently reported being less exclusively heterosexual, and regardless of political alignment, participants reported less certainty about their sexual orientation, relative to controls. In Study 2, after exposure to fluid or continuous manipulations heterosexual participants were up to five times more likely than controls to rate themselves as non-exclusively heterosexual. Additionally, those in the continuous condition reported less certainty about their sexual orientation and were more willing to engage in future same-sex sexual experiences, than those in the control condition. These results suggest that non-traditional theories of sexual orientation can lead heterosexuals to embrace less exclusive heterosexual orientations.

People hold a variety of beliefs about the nature and origins of sexual orientation^1–3^. Traditionally, sexual orientation has been viewed as existing in discrete categories (e.g., homosexual vs. heterosexual) and as being stable over one’s life. In recent years, the idea that sexual orientation exists on a continuum from homosexual to heterosexual with varying degrees of bisexuality in-between (*continuity*) and that one’s preference for men versus women can shift overtime and context (*sexual fluidity*) has been on the ascendancy within academia, LGBTIQ + activism, and popular culture. From Miley Cyrus coming out as “sexually fluid”^4^ to TIME magazine running a feature on men who are “Not Straight. Not Bisexual. [But] Mostly Heterosexual”^5^ non-traditional ways of conceptualizing sexual orientation are now mainstream. Reflecting this trend, a recent YouGov poll found that Americans are now evenly split on whether they view sexual orientation as a continuum (40%) versus as a gay/straight binary (42%)^6^—suggesting the notion of the sexual orientation continuum has now permeated the broader culture, 70 years on from Alfred Kinsey first introducing the Kinsey Scale^7^.

Why do these beliefs matter? A body of research indicates that people’s beliefs about sexual orientation are predictive of their attitudes toward sexual minorities. Typically, it is found that believing homosexuality to be biologically determined and immutable, rather than chosen or malleable, is associated with more acceptance of sexual minorities, presumably because gay/lesbian individuals are seen as less blameworthy for their sexualities^8–11^. On the other hand, viewing sexual orientation as existing in binary gay/straight categories predicts more prejudice, perhaps because it leads gay/lesbian individuals to be seen as more fundamentally different^8,10^. Likewise, denying the existence of bisexuality is a core component of the unique stigma (or *biphobia*) faced by bisexual folk^12^.

While considerable attention has been paid to how our beliefs about sexual orientation may contribute to, or ameliorate sexual prejudice, little attention has been paid to how sexual orientation beliefs may shape understanding of our own sexuality. Making sense of our sexual orientation can be a difficult and confusing process—particularly when we possess stigmatised same-sex feelings. Although apprehending the nature of our erotic interests might seem like a straightforward process, how we come to understand and label our sexuality is at least partly circumscribed by the concepts and language available to us. The proliferation of non-traditional sexual identities in recent years appears to be a testament to this^13^.

Relevant to this conversation, YouGov polling finds that the proportion of Americans identifying as completely heterosexual (Kinsey 0) dropped 9% between 2015 (78%) and 2018 (69%). This shift is most dramatic among 18-to-34-year-olds-where exclusive heterosexuals now comprise a slim majority of the population (55%)^6^. Similar trends have been observed in other Western nations^14^. The bulk of those reporting same-sex attraction are non-exclusive heterosexuals (those mostly attracted to the opposite-sex but with some small degree of same-sex attraction)—a group who is more prevalent than all other same-sex attracted individuals combined^15^. This marked increase in men and women reporting a non-exclusive heterosexual orientation is surely partly attributable to dramatic societal progress in the acceptance of same-sexuality in recent years^16^. However, could it also be the case that exposure to continuous or fluid theories of sexual orientation—might provide some heterosexuals a framework to acknowledge and embrace their occasional same-sex attractions for the first time?

There is a reason to expect that providing information couched in scientific content/language may increase the perceived value of the information and increase the likelihood of changes in beliefs. Theoretical accounts in the persuasion literature suggest that individuals may apply two different processing approaches in their receptivity to new information. Both the Heuristic Model of Persuasion^17^ and the Elaboration Likelihood Model of Persuasion^18^ identify elements, such as expertise, scientific framing, and source credibility as features that play an important role in leading people to adopt new—or adjust old—beliefs, independent of the actual persuasiveness of the argument being made under certain conditions. Experimental^19^ and neurological^20^ findings support this robust phenomenon.

In addition to the potential effect of the source of the information, it seems likely that certain types of individuals will be more influenced by exposure to continuous or fluid theories of sexual orientation than others. A significant body of literature demonstrates that female sexual orientation is more continuous and fluid than male sexual orientation^21,22^. For instance, women are significantly more likely to identify as bisexual or mostly heterosexual, than men^15,23^. To the extent that continuous and fluid notions more accurately describe women’s sexual orientation—it would be expected that they would be readily accepted by women than men. Additionally, as women tend to report more positive attitudes toward same-sex sexuality than men^24^, and face less social backlash for same-sex interests and sexual behaviour^25^, they would be expected to be more willing to embrace some degree of same-sex attraction if it were discovered. Indeed, continuous and fluid notions of sexual orientation may be particularly threatening to heterosexual males given that male-same sex sexuality violates male gender role norms and constitute a challenge to their privileged manhood/masculinity^26^. A significant body of literature demonstrates that males experience anxiety when faced with threats to their masculinity and engage in efforts to re-assert their masculine status^27^. Relatedly, heterosexual males more so than heterosexual females, are motivated to maintain rigid distinctions between themselves and homosexual individuals of the same gender—and express more homophobic attitudes when this distinctiveness is threatened^28^—in order to assert their heterosexuality. In sum, these findings lead us to predict that heterosexual males will be less likely to embrace continuous or fluid theories of sexual orientation than heterosexual females.

Next, politically progressive individuals may be more affected by exposure to continuous and fluid accounts than conservative individuals. Research has demonstrated that political conservatives tend to perceive social categories in a more discrete manner than progressives, including sexual orientation^8^. This is likely associated with traits such as need for cognitive closure and intolerance of ambiguity/uncertainty which are found to be higher in conservatives than progressives^29^. Moreover, conservatives tend to be more prejudicial toward LGBT individuals^30^. Presumably this would lead to greater reluctance in acknowledging their own same-sex attraction (if present) and reduced tendency to accept a non-exclusive heterosexual orientation.

We decided to test the above possibilities experimentally. Across two studies we examined whether exposure to different accounts of sexual orientation (continuous or discrete in Study 1, and continuous, discrete, fluid, or stable in Study 2) influenced how heterosexual men and women rated their sexual orientation on a Kinsey-type continuum, as well as their willingness to engage in sexual/romantic encounters with members of the same-sex, and their level of uncertainty in their sexual orientation. Additionally, both gender and political orientation were examined as potential moderators of these effects. We hypothesized:

1. Those participants exposed to continuous or fluid theories of sexual orientation will be more likely to report non-exclusive heterosexual orientations, sexual orientation uncertainty, and willingness to engage in same-sex sexual/romantic experiences, than those in the control group.
2. The effect of continuous and fluid theories of sexual orientation on sexual orientation indices will be moderated by (a) gender and (b) political orientation, such that women and political progressives will be more likely to report non-exclusive heterosexuality, sexual orientation uncertainty, and willingness to engage in same-sex sexual/romantic experiences, than men and conservatives respectively.
3. Those participants exposed to discrete, or stable theories of sexual orientation will be less likely to report non-exclusive heterosexual orientations, sexual orientation uncertainty, and willingness to engage in same-sex sexual/romantic experiences, than those in the control group. We undertook exploratory analyses to examine if gender or political orientation moderated these effects.

These hypotheses and planned analyses were pre-registered online (https://osf.io/2chd5/?view_only=f07db63b93274334ae5786c0b2b17bb0).

## Study 1

### Methods

Participants were recruited from a first-year psychology participation pool at The University of Sydney. To be eligible for participation, students were required to speak fluent English, identify as heterosexuals (“straight”) on a pre-screener survey, and identify with the gender they were assigned at birth. The final sample consisted of 180 participants (120 female). Participants ranged in age from 18 to 53 (*M* = 19.51 years; *SD* = 3.76). Sample size was determined before any data analysis as outlined in the OSF pre-registration. With regards to ethnicity, 46.2% identified as Asian, 44% as White, 5.4% as Middle-Eastern, 3.8% as mixed ethnicity, and 0.5% as Indigenous Australian. All participants reported their sexual identity as “straight (heterosexual)”. Based on a 9-point Kinsey-type scale completed at pre-test, 132 participants (82 female; 76.1% total) rated their sexual orientation as exclusively heterosexual [i.e., “1”], 40 (37 female; 22.3% total) as almost exclusively heterosexual [i.e., “2”], and 3 (1 female; 1.6% total) as mostly heterosexual [i.e., “3”]. Political orientation was rated on a 7-point scale, from Extremely Conservative [1] to Extremely Progressive [7]; 60.9% of participants identified their political orientation as being between fairly progressive [5] and extremely progressive [7], 12% as between extremely conservative [1] and fairly conservative [3], and 27.1% as moderate [4]. Between-subjects analyses of variance (ANOVAs) revealed no significant differences in age, gender, political orientation, or sexual orientation at pre-test across conditions (*ps* > 0.05).

#### Procedure

The study was completed online and was described as a study examining “Attitudes & Sexual Prejudice”. After providing informed consent, participants completed a demographics survey, including a pre-test measure of their sexual orientation. Then, participants were randomly assigned to one of three conditions: continuous, discrete, or control. In the continuous condition, participants read a one-page article detailing recent evidence that sexual orientation exists on a continuum inclusive of mostly heterosexual orientations. In the discrete condition, the article presented evidence that sexual orientation exists in three discrete forms, heterosexuality, bisexuality, and homosexuality. The third condition (control condition) was a popular science article on the effects of global warning. After reading the article, participants were asked five true or false questions based on the information they had just read (i.e., a manipulation check). These were to ensure that participants had read and comprehended the concept being described (e.g., participants in the discrete condition were asked questions such as “Evidence at present supports the idea that sexual orientation does exist in three discrete categories, heterosexual vs. homosexual vs. bisexual” to check they had apprehended this notion). Participants then re-rated their sexual orientation (on the same pre-test measure) and responded to questions assessing their certainty about their sexual orientation and their willingness to engage in same-sex sexual or romantic behaviors.

#### Materials

##### Demographics

Participants indicated their age, ethnicity, religiosity, political orientation, birth sex, present gender identity, and sexual orientation.

##### Sexual orientation

A 9-point Kinsey-type continuum^31^, was used to assess sexual orientation (1 = *exclusively heterosexual*, 2 = *almost exclusively heterosexual*, 3 = *mostly heterosexual*, 4 = *heterosexual-leaning bisexual*, 5 = *bisexual*, 6 = *homosexual-leaning bisexual*, 7 = *mostly homosexual*, 8 = *almost exclusively homosexual*, 9 = *exclusively homosexual*). Participants were asked to indicate “what sexual orientation identity [they] identified with at present?” at both pre and post-test.

##### Sexual orientation uncertainty

Sexual orientation uncertainty was measured using the sexual orientation uncertainty subscale (4 items) of the Lesbian Gay Bisexual Identity Scale^32^ with items adapted for heterosexuals. Items in this subscale measured the extent to which people were uncertain about what their sexual orientation was (e.g., “*I’m not totally sure what my sexual orientation is*”). All items were measured on a 4-point Likert-type scale ranging from 1 (*strongly disagree*) to 4 (*strongly agree*). Higher scores were indicative of more uncertainty about one’s sexual orientation. Good internal consistency was demonstrated for this subscale (α = 0.77).

##### Willingness to experience same-sex sexual/romantic encounters

A 4-item scale was constructed to assess an individuals’ willingness to engage in sexual/romantic relations with people of the same-sex. Items assessed the willingness of participants to have a crush on, fall in love with, have a romantic relationship with, and kiss a member of the same-sex (i.e., women were asked about their willingness to engage in sexual/romantic encounters with other women [e.g., “*how willing are you to kiss a woman*”], whereas men were asked about their willingness to engage in sexual/romantic encounters with other men [e.g., “*how willing are you to kiss a man*”]). Responses were recorded using a 5-point Likert-type scale (1 = *very unwilling*, 3 = *somewhat willing*, 5 = *extremely willing*). Higher scores indicted greater willingness to engage in sexual/romantic interactions with members of the same-sex. The scale demonstrated excellent internal consistency (α = 0.93).

##### Experimental manipulation

The wordings used in the continuous and discrete conditions were closely matched—with both using the same template—but with specific aspects of sentences changed to reflect either sexual orientation as existing on a continuum or as existing in three discrete categories (heterosexual, bisexual, or homosexual). The control condition was not matched for content (it comprised a popular science article on the effects of climate change in the high arctic). It was matched for length. See Supplementary Information for manipulations used.

#### Data preparation

To examine the effect of continuous and discrete conditions on sexual orientation, the conditions were dummy coded, with the control condition serving as the baseline comparison. Changes in sexual orientation were operationalised as the difference in self-rating before and after the manipulation (i.e., a positive difference-score represented a shift toward a more same-sex sexual orientation, whereas a negative difference-score a shift toward a more opposite-sex sexual orientation). Because responses to the sexual orientation uncertainty and willingness scales were extremely non-normal in both studies (expected given the heterosexual sample), responses were dichotomized (i.e., strongly disagree/not at all willing = 0; somewhat disagree to strongly agree/somewhat willing to extremely willing = 1).

#### Data analyses

For each dependent variable (DV: sexual orientation, sexual orientation uncertainty, willingness to engage in same-sex sexual/romantic behaviors), regression models took the same form. In Step 1, we examined the main effect of the dummy-coded continuous versus control variable, and the dummy-coded discrete versus control variable, on the dependent variable of interest. In Step 2 (which was only interpreted if the R-Square change for Step 2 was significant), we examined the main effect of political orientation (in continuous form), gender (male versus female), and added interactions between political orientation × gender, continuous condition × political orientation, continuous condition × gender, discrete condition × political orientation, and discrete condition × gender. Hierarchical Linear Regressions were used to analyse data for the sexual orientation difference-scores. Binomial Logistic Regressions were used to analyse sexual orientation uncertainty and willingness to engage in sexual/romantic interactions with the same-sex, given the binary nature of these dependent variables. Where significant interactions were observed, we report post-hoc simple slopes analyses using Hayes’s PROCESS Macro, version 3.0^33^. To reduce the likelihood of committing Type-I errors, a Bonferroni correction was applied to account for the multiple DVs. Given that we examined three DVs, we used a *p*-value of 0.0167 (i.e., 0.05/3) to determine statistical significance.

### Results and discussion

#### Sexual orientation self-rating

**Figure 1:**
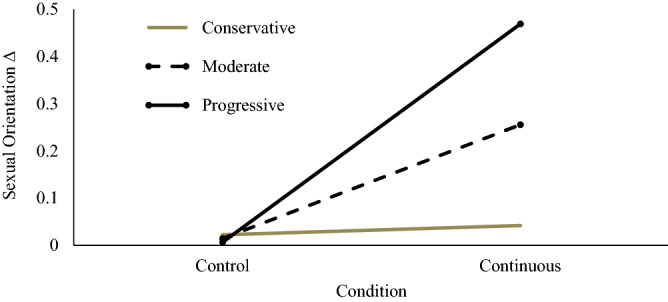
Change in sexual orientation self-rating as a function of condition (*control* or *continuous*) and political orientation (*conservative, moderate*, or *progressive*). Higher scores indicate a greater shift in sexual orientation from pre- to post-manipulation.

The main hypothesis, that those who read the continuous account of sexual orientation would shift their ratings of their own sexual orientation to be less exclusively heterosexual (relative to those in the control group: Hypothesis 1), was tested first. Although no main effects for the continuous or discreteness manipulation were present, political orientation significantly moderated the effect of the exposure to the continuous condition on sexual orientation self-ratings (so significant interactions were present for the discreteness condition), supporting Hypothesis 2B. We tested simple slopes to examine at what values of political orientation (− 1 SD below the mean, at the mean, + 1 SD above the mean) the continuous manipulation significantly affected sexual orientation change. As there was no main effect of gender and no significant condition × gender interaction (contrary to Hypothesis 2A), gender variables were not included as covariates when examining simple slopes. Post-hoc simple slopes analyses indicated that while politically progressive individuals demonstrated shifts in sexual orientation self-ratings following exposure to the continuous condition, *t*(176) = 3.410, *β* = 0.462, *p* < 0.001, CI [0.19, 0.73], and a similar effect was observed among political moderates, *t*(176) = 2.475, *β* = 0.241, *p* = 0.014, CI [0.05, 0.43], political conservatives showed no such an effect *t*(176) = 0.180, *β* = 0.02, *p* = 0.857, CI [− 0.19, 0.23]. No other interactions were significant (see Table 1 and Fig. 1).

**Table 1:** Study 1. Effects of reading continuous or discrete accounts on sexual orientation self-ratings and secondary indices.

Variable	Sexual Orientation ∆				Willingness to have Same Sex Experiences			Sexual Orientation Uncertainty		
	β	95% CI	*sr*^2^		β	95% CI	Exp(β)	β	95% CI	Exp(β)
Model 1				Model 1						
*R*^2^	0.020			Step χ^2^(2)	2.588			5.731		
*F* Change	1.837			Nagelkerke *R*^*2*^	0.019			0.044		
Continuous	0.166	[− 0.007, 0.439]	0.142		− 0.458	[0.301, 1.332]	0.633	0.871*		2.389
Discrete	0.076	[− 0.118, 0.307]	0.066		0.107	[0.543, 2.281]	1.113	0.126		1.135
Model 2				Model 2						
*R*^2^	0.126**			Step χ^2^(9)	28.03***			8.45		
*F* change	2.936**			Nagelkerke *R*^*2*^	210			0.106		
Gender	0.032	[− 0.323, 0.404]	0.016		1.474*	[1.151, 16.562]	4.366	− 0.417	[0.142, 3.054]	0.659
Political orientation	− 0.267	[− 0.277, 0.026]	− 0.117		0.237	[0.700, 2.294]	1.268	0.275	[0.685, 2.534]	1.317
Continuous	0.143	[− 0.248, 0.533]	0.052		− 0.085	[0.215, 3.926]	0.919	0.624	[0.430, 8.087]	1.865
Discrete	0.187	[− 0.204, 0.579]	0.068		0.015	[0.241, 4.282]	1.015	− 0.179	[0.177, 3.958]	0.836
Gender × PO	0.276*	[0.023, 0.312]	0.164		0.214	[0.713, 2.152]	1.239	0.443	[0.874, 2.776]	1.558
Continuous × PO	0.31**	[0.094, 0.442]	0.218		− 0.038	[0.491, 1.889]	0.963	− 0.452	[0.310, 1.305]	0.636
Discrete × PO	0.016	[− 0.153, 0.180]	0.011		− 0.154	[0.447, 1.644]	0.858	− 0.309	[0.347, 1.554]	0.734
Continuous × gender	0.169	[− 0.217, 0.744]	0.078		− 0.276	[0.129, 4.462]	0.759	0.780	[0.329, 14.438]	2.180
Discrete × gender	− 0.066	[− 0.564, 0.382]	− 0.027		0.229	[0.220, 7.192]	1.258	0.629	[0.264, 13.340]	1.876

*PO* political orientation.

*p < 0.05; **p < 0.01; ***p < 0.001.

Were these shifts in sexual orientation ratings confined to those who already reported a non-exclusive sexual orientation at pre-test, or did those who reported an exclusive heterosexual orientation shift to reporting a non-exclusive heterosexual orientation after reading the continuous account? To test this, the following elements were added to the regression model: the main effect of exclusivity at pre-test (0 = exclusive heterosexual, 1 = non- exclusively heterosexual), two-way interactions between exclusivity × condition (continuous or discrete), and three-way interactions between exclusivity × political orientation × condition (continuous or discrete). This unregistered, exploratory analyses, then tested whether the significant two-way political orientation × continuous condition interaction, was further moderated by sexual orientation at pre-test. No significant three-way interaction was observed, *t*(165) = − 2.137, *β* = − 0.186, *p* = 0.034, although a trend was present. Follow-up analyses indicated sexual orientation shifts were significant among those who were exclusively heterosexual, *t*(127) = 3.216, *β* = 0.348, *p* = 0.002, and non-significant among those who were non-exclusively heterosexual, *t*(33) = 0.920, *β* = 0.281, *p* = 0.364, at pretest (but note that the samples substantially differ in size). The effect of discreteness on sexual orientation self-ratings was not moderated by the exclusivity of one’s heterosexuality at pre-test, *t*(179) = 0.499, *β* = 0.060, *p* = 0.618.

#### Secondary indices

Secondary indices of sexual orientation demonstrated convergent evidence for changes in how people conceived of their sexual orientation among those exposed to the continuous condition, supporting Hypothesis 1. A main effect indicated that those in the continuous condition were less certain about their sexual orientation than controls. On the other hand, exposure to the discreteness manipulation did not significantly impact certainty about one’s sexual orientation failing to support Hypothesis 3. Contrary to predictions, participants’ willingness to engage in same-sex sexual/romantic behaviors was not significantly affected by exposure to the manipulations (Table 1 for analysis, Tables 2 and 3 for descriptive data; and Fig. 3A,B).

**Table 2:** Study 1. Post-manipulation ratings of sexual orientation uncertainty and willingness for same-sex encounters in control, continuous, and discrete conditions.

	Sexual orientation uncertainty		Willingness for same-sex encounters	
	Strongly disagree	Somewhat disagree–strongly agree	Not at all willing	Somewhat willing–extremely willing
Control	74.1% (43)	25.9% (15)	41.4% (24)	58.6% (34)
Continuous	54.5% (30)	45.5% (25)	52.7% (29)	47.3% (26)
Discrete	71.6% (48)	28.4% (19)	38.8% (26)	61.2% (41)

**Table 3:** Study 1. Pre and post manipulation ratings of sexual orientation and sexual orientation change.

	Exclusively heterosexual	Almost exclusively heterosexual	Mostly heterosexual
**Pre-manipulation**			
Control	70.7% (41)	27.6% (16)	1.7% (1)
Continuous	72.7% (40)	23.6% (13)	3.6% (2)
Discrete	83.6% (56)	16.4% (11)	0% (0)
**Post-manipulation**			
Control	75.9% (44)	20.7% (12)	3.4% (2)
Continuous	65.6% (36)	30.9% (17)	3.6% (2)
Discrete	77.6% (52)	22.4% (15)	0.0% (0)
**Change (pre–post manipulation)**			
Control	+ 5.2% (3)	− 6.9% (4)	− 1.7% (1)
Continuous	− 6.6% (4)	+ 6.3% (4)	0.0% (0)
Discrete	− 6.0% (4)	+ 6.0% (4)	0.0% (0)

Percentages in the “Change (Pre–Post manipulation)” represent changes in the proportion of participants endorsing “exclusively heterosexual”, “almost exclusively heterosexual”, and “mostly heterosexual” for each condition (Control, Continuous, Discrete) post manipulation.

## Study 2

In sum, Study 1 found that students exposed to continuous theories of sexual orientation reported less exclusive heterosexual orientations thereafter (with the exception of politically conservative students), and regardless of political orientation, those exposed to continuous theories reported less certainty about their sexual orientation. Study 2 attempted to replicate and extend the findings of Study 1. First, to pacify concerns that our continuous manipulation may not have adequately primed continuity, it was revised to emphasize the existence of potentially infinite gradations of sexual orientation between exclusively heterosexual and exclusively homosexual, rather than merely the existence of additional intermediate categories (i.e., mostly heterosexual and mostly gay) as described in the continuous manipulation in Study 1. Second, additional informational accounts of sexual orientation (fluid versus stable) were introduced to explore their effects on self-perceived sexual orientation. Third, we recruited a more nationally representative community sample with regards to age, political orientation, and gender. Finally, sexual orientation was only assessed post manipulation, due to concerns that participants may strive for consistency in their pre/post manipulation ratings of their sexual orientation, reducing the strength of sexual orientation change in self-reports. As such rather than assessing change in reporting of sexual orientation following manipulation, we examined post-manipulation differences in reported sexual orientation in continuous, discrete, fluid, and stable conditions compared to control.

## Methods

### Participants

A nationally representative sample was recruited via Qualtrics panels (participants were reimbursed by Qualtrics for their time). The final sample consisted of 460 (232 female) cisgender heterosexual identified participants (after excluding 48 participants who failed the experimental manipulation check). Sample size was determined before any data analysis as outlined in the OSF pre-registration. Ages ranged from 18 to 83 (*M* = 45.65, *SD* = 15.74); 78.7% of the sample identified as White, 13.5% identified as Asian, 1.3% identified as Indigenous Australian, and the remaining 6.5% reported a range of ethnicities. The demographic break-down of participants with regard to gender, age, and ethnicity is in line with the national values^34^. Regarding political orientation, 31.3% identified somewhere between extremely conservative and fairly conservative, 33.5% as moderate, 35.2% as somewhere between fairly progressive and extremely progressive. All participants used the label “straight” to describe their sexual orientation.

### Procedure

As with Study 1, participants gave their informed consent online before beginning the study, which was advertised as examining “Attitudes & Sexual Prejudice”. After completing the demographics, participants were randomly assigned to one of the five conditions. The continuous and discrete manipulations presented the same concepts as in Study 1, whereas Study 2 added two additional conditions—one emphasizing how sexual orientation can change over time (fluid condition), and the other highlighting how sexual orientation remains fixed across time (stable condition). The control condition was a popular science article describing the anatomy of trees. Following the manipulation, participants answered three true or false questions based on the information they had just read (manipulation check) and completed the sexual orientation measures. Participants then completed the same set of outcome measures as in Study 1 (those relevant to this study were sexual orientation, sexual orientation uncertainty, and willingness to engage same-sex behaviours). They were then reimbursed for their participation and debriefed.

### Materials

#### Demographics

The same demographics were collected in Study 2 as in Study 1, apart from sexual orientation, which was only assessed at post-test due to concerns that participants would recall their pre-test response and strive for consistency in self-ratings.

#### Sexual orientation identity

The same 9-point question from Study 1 was used to assess sexual orientation. Mean post-test sexual orientation rating was intermediate exclusively heterosexual and almost exclusively heterosexual (*M* = 1.36, *SE* = 0.96).

#### Sexual orientation uncertainty

As with Study 1, sexual orientation uncertainty was measured using the 4-item sexual orientation uncertainty subscale of the Lesbian Gay Bisexual Identity^32^ adapted for heterosexuals. Good internal consistency was demonstrated for this subscale (α = 0.82).

#### Willingness to experience same-sex encounters

Willingness to experience same-sex encounters was measured using the scale designed for Study 1. Excellent internal consistency was demonstrated for this scale (α = 0.94).

#### Manipulations

As mentioned above, the continuous manipulation was revised for Study 2, due to a concern that the continuous condition in Study 1 did not prime the concept of the sexual orientation continuum strongly enough. The discreteness manipulation was also revised to match the length and style of the new continuous manipulation. The fluid and stable conditions were matched with each other, but not with the continuous and discreteness condition (which, as in Study 1, were matched with one another). The fluidity condition defined the concept of sexual fluidity and summarized scientific evidence purporting to demonstrate that sexual orientation may shift across the life-course, sometimes multiple times (making it explicit that such shifts are typically outside of individual conscious control and should not be confused with choice). The stability condition defined the concept of sexual stability and presented evidence that sexual orientation is fixed once it emerges early in life. Conceptually, matching all four manipulations (continuous, discrete, fluid, stable) was not possible as the ideas presented in the fluid and stable conditions did not naturally reflect the ideas of sexual orientation as categorical or continuous. Manipulations used can be found in supplemental materials.

### Data preparation

In the final sample, 89 participants (44 female) were randomly assigned to the continuous condition, 88 (43 female) to the discrete condition, 92 (49 female) to the fluid condition, 89 (45 female) to the stable condition, and 102 (51 female) to the control group. Between-subjects ANOVAs revealed no significant differences between the conditions on age, gender, or political orientation (*ps* > 0.05). Each condition was dummy coded, using the control condition as the reference group. The responses to the uncertainty and willingness scales were extremely non-normal and were subsequently dichotomized in the same manner as in Study 1. Two participants, one in the control condition and one in the discrete condition, reported their sexual orientation as exclusively homosexual on the post-test 9-point Kinsey type scale. These participants were excluded from the relevant analysis of sexual orientation due to high likelihood these were non-serious responses (meaning that sample size for this analysis was n = 458, but still n = 460 for subsequent analysis of sexual orientation uncertainty and willingness to engage in same-sex experiences).

### Data analyses

Similar to Study 1, for each dependent variable of interest the regression models took the same form. The dummy coded experimental conditions contrasted with the control condition were entered in the first step. Step 2 added the main effects of political orientation and gender and the interactions between political orientation × gender, political orientation and experimental conditions (continuous, discrete, fluid, stable), and between gender and experimental conditions (continuous, discrete, fluid, stable). As in Study 1, Binomial Logistic Regression was used to analyse sexual orientation uncertainty and willingness to engage in sexual/romantic interactions with the same-sex. As in Study 1, due to multiple comparisons, a Bonferroni correction was applied to each variable, such that a *p*-value of 0.167 was used to determine statistical significance. Although Bonferroni is considered a conservative correction, given our relatively large sample size (for experimental social psychology) we felt this would minimize the chance of spurious findings.

### Ethics

The experimental protocol was conducted in accordance to guidelines of The University of Sydney Research Ethics Committee. The experimental protocol (for research involving humans) was approved by The University of Sydney Research Ethics Committee.

## Results

**Figure 2:**
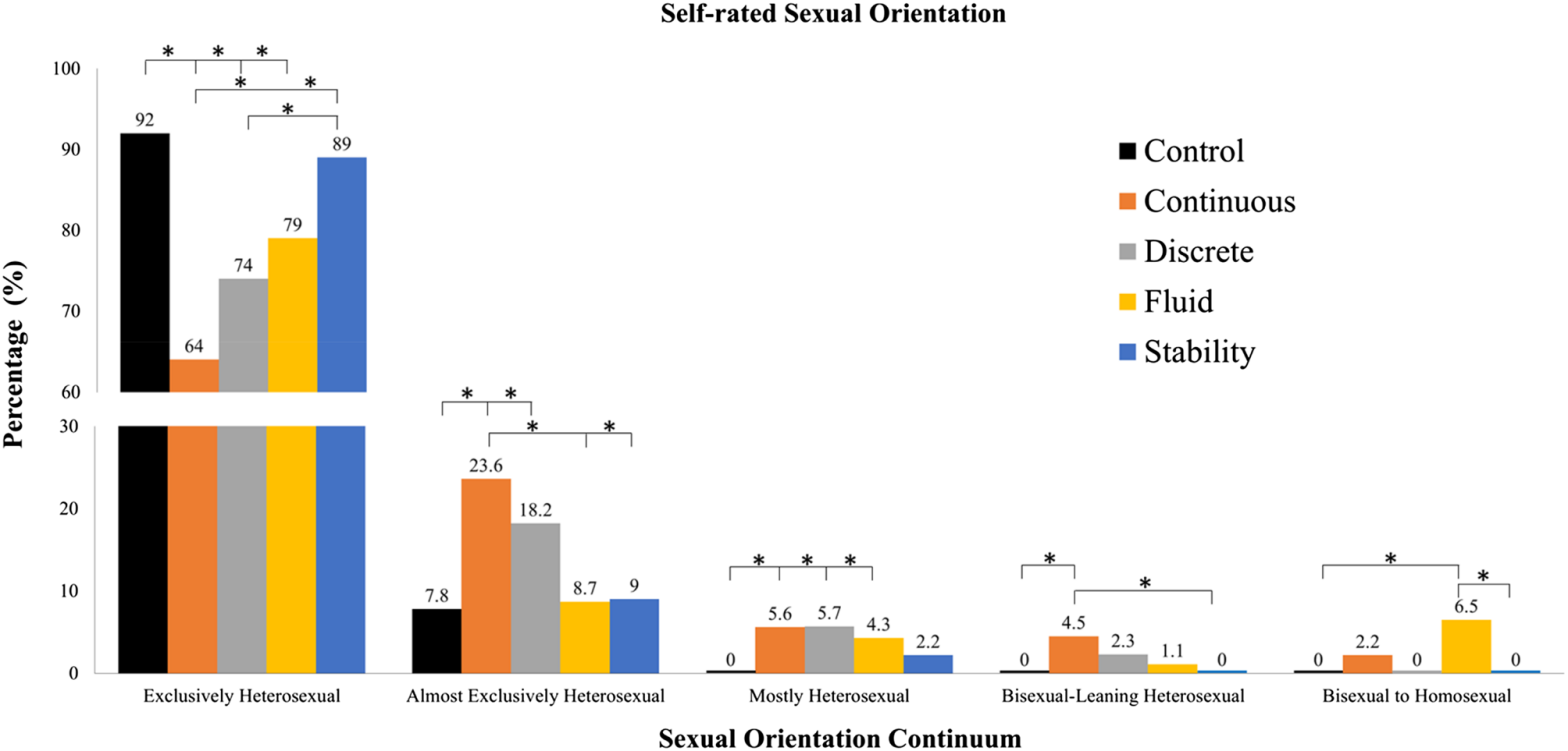
Sexual orientation self-ratings from Exclusively Heterosexual through to Exclusively Homosexual after reading a control article or a continuous, discrete, fluid, or stable account of sexual orientation. Differences in sexual orientation *p < 0.05.

In study 2, in line with our primary hypotheses (Hypothesis 1), those who read the continuous or fluid accounts were more likely to select a non-exclusive heterosexual orientation (36% and 20.7% respectively) than those who read the control article (7.8%). Unlike in Study 1, contrary to Hypothesis 2B, the effect of the continuous manipulation on sexual orientation was not significantly moderated by political orientation. Those exposed to the discrete account demonstrated a non-significant trend toward being more non-exclusively heterosexual (26.1% reported non-exclusive heterosexual attraction patterns) than controls (contrary to Hypotheses 3 that discreteness beliefs would actually lead to greater exclusivity). Also counter to Hypotheses 3, exposure to the stable account did not impact sexual orientation ratings (12.2% reported non-exclusive heterosexual attraction patterns). Contrary to Hypothesis 2A, gender failed to significantly moderate the effect of continuous and fluid manipulations on self-reported sexual orientation. See Tables 4 and 5 and Fig. 2.

**Table 4:** Study 2. Effects of reading continuous, discrete, fluid, and stable accounts on sexual orientation self-ratings and secondary indices.

Variable	Sexual orientation post-test				Willingness to have same sex sexual/romantic experiences			Sexual orientation uncertainty		
	β	95% CI	*sr*^*2*^		β	95% CI	Exp(β)	β	95% CI	Exp(β)
Model 1				Model 1						
*R*^2^	0.059***			Step χ^2^(4)	8.301***			13.428**		
*F* Change	7.111			Nagelkerke *R*^*2*^	0.025***			0.041**		
Continuous	0.239***	[0.266, 0.722]	0.194		0.794**	[1.219, 4.014]	2.212	1.122**	[1.598, 5.901]	3.071
Discrete	0.138*	[0.059, 0.518]	0.113		0.270	[0.710, 2.416]	1.310	0.717*	[1.046, 4.010]	2.048
Fluid	0.211***	[0.206, 0.658]	0.171		0.233	[0.689, 2.312]	1.262	0.834*	[1.192, 4.445]	2.302
Stable	0.027	[− 0.172, 0.284]	0.022		0.083	[0.585, 2.016]	1.086	0.466	[0.804, 3.159]	1.594
Model 2				Model 2						
*R*^2^	0.543			Step χ^2^(9)	52.151***			14.495		
*F* Change	0.897			Nagelkerke *R*^*2*^	0.170			0.084		
Gender	0.027	[− 0.269, 0.358]	0.013		0.802	[0.908, 5.481]	2.231	− 0.408	[0.242, 1.827]	0.665
Political Orientation	− 0.014	[− 0.145, 0.128]	− 0.006		0.339	[0.939, 2.096]	1.403	− 0.088	[0.597, 1.404]	0.916
Continuous	0.207*	[0.266, 0.722]	0.194		1.090	[1.199, 7.379]	2.975	0.487	[0.540, 3.647]	5.156
Discrete	0.123	[0.059, 0.518]	0.113		0.269	[0.485, 3.537]	1.309	0.666	[0.472, 3.231]	3.647
Fluid	0.171*	[0.206, 0.658]	0.171		− 0.346	[0.244, 2.056]	0.708	0.387	[0.593, 3.857]	3.231
Stable	0.027	[− 0.172, 0.284]	0.022		− 0.514	[0.199, 1.797]	0.598	0.429	[0.242, 1.827]	30.857
Gender × PO	0.051	[− 0.068, 0.153]	0.034		0.022	[0.737, 1.418]	1.023	0.060	[0.774, 1.457]	1.457
Continuous × PO	− 0.004	[− 0.182, 0.172]	− 0.002		− 0.210	[0.500, 1.312]	0.810	− 0.169	[0.501, 1.424]	0.845
Discrete × PO	0.089	[− 0.056, 0.307]	0.062		0.381	[0.832, 2.575]	1.464	0.523	[0.961, 2.965]	1.688
Fluid × PO	− 0.076	[− 0.266, 0.077]	− 0.050		− 0.149	[0.524, 1.417]	0.862	0.192	[0.722, 2.033]	1.211
Stable × PO	0.003	[− 0.178, 0.186]	0.002		0.033	[0.598, 1.786]	1.033	0.046	[0.604, 1.816]	1.048
Continuous × gender	0.043	[− 0.342, 0.580]	0.023		− 0.553	[0.167, 1.979]	0.575	0.839	[0.609, 8.789]	2.314
Discrete × gender	0.028	[− 0.382, 0.538]	0.015		− 0.111	[0.238, 3.369]	0.895	0.705	[0.508, 8.060]	2.023
Fluid × gender	0.051	[− 0.318, 0.589]	0.027		0.897	[0.642, 9.369]	2.453	1.192	[0.856, 12.681]	3.294
Stable × gender	− 0.006	[− 0.477, 0.443]	− 0.003		0.704	[0.515, 7.934]	2.022	0.136	[0.286, 4.595]	1.146

*PO* political orientation

*p < 0.05; **p < 0.01; ***p < 0.001.

**Table 5:** Study 2. Post-manipulation ratings of sexual orientation, sexual orientation uncertainty and willingness for same-sex encounters in control, continuous, discrete, fluid, and stable conditions.

Condition	Sexual orientation					Sexual orientation uncertainty		Willingness for same-sex encounters	
	Exclusively heterosexual	Almost exclusively heterosexual	Mostly heterosexual	Bisexual-leaning heterosexual	Bisexual to mostly homosexual	Strongly disagree (1)	Somewhat disagree (2)–strongly agree (4)	Not at all willing (1)	Somewhat willing (2)–extremely willing (5)
Control	92.2% (94)	7.8% (8)	0.0% (0)	0.0% (0)	0.0% (0)	80.4% (82)	19.6% (20)	70.6% (72)	29.4% (30)
Continuous	64.0% (57)	23.6% (21)	5.6% (5)	4.5% (4)	2.2% (2)	58.4% (52)	41.6% (37)	51.7% (46)	48.3% (43)
Discrete	73.9% (65)	18.2% (16)	5.7% (5)	2.3% (2)	0.0% (0)	68.2% (60)	31.8% (28)	64.8% (57)	35.2% (31)
Fluid	79.3% (73)	8.7% (8)	4.3% (4)	1.1% (1)	6.5% (6)	65.2% (60)	34.8% (32)	65.2% (60)	34.8% (32)
Stable	88.8% (79)	9.0% (8)	2.2% (2)	1.1% (1)	6.5% (6)	73.0% (65)	27.0% (24)	68.5% (61)	31.5% (28)

**Figure 3:**
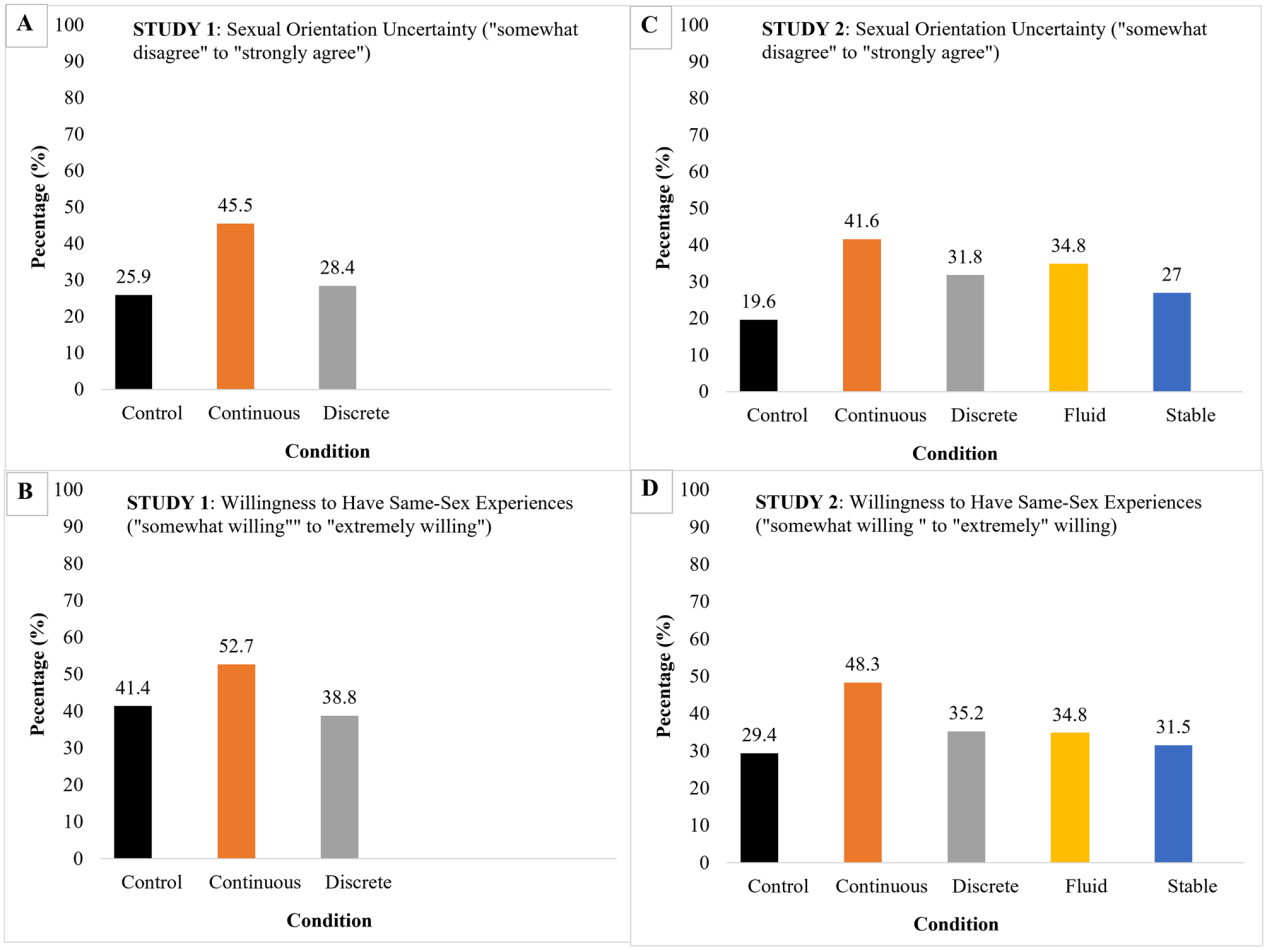
Post-manipulation sexual orientation uncertainty and willingness to have same-sex experiences in Study 1 and Study 2. For (**A**) and (**B**), Y-axis represents the percentage of participants in each condition who reported some-level of sexual orientation uncertainty (somewhat disagree [2] to strongly agree [4]). For (**C**) and (**D**), Y-axis represents the percentage of participants in each condition who report some level of willingness to have same-sex experiences (somewhat [2] to extremely willing [5]).

Providing convergent evidence for continuity and fluidity beliefs influencing self-perceived sexual orientation, those exposed to the continuous condition demonstrated greater uncertainty about their sexual orientation than controls (41.6% vs. 19.6%) and a non-significant trend in the same direction was observed among those who were exposed to the fluid condition (34.8%) (partially supporting Hypotheses 1). Neither those in the discrete condition (31.8%) nor stable condition (27%) demonstrated less certainty relative to controls (27%). Finally, only those exposed to the continuous condition reported greater willingness to engage in same-sex sexual/romantic experiences in the future, relative to those in the control condition (48.3% vs. 29.4%) further supporting Hypothesis 1. Other manipulations failed to demonstrate an effect for willingness to engage in same-sex sexual/romantic experiences (Table 2 and Fig. 3C,D). Again, contrary to our Hypotheses 2A, gender did not moderate any of these effects significantly.

## General discussion

These studies show that how heterosexual individuals perceive their sexual orientation can be influenced by exposure to different theories regarding the nature of sexual orientation. In Study 1, exposure to continuous notions of sexual orientation caused political progressive and moderate, but not conservative university students to change how they perceived their sexual orientation to a non-exclusive heterosexual orientation (in support of Hypothesis 2B)—presumably as young progressives were less resistant to accepting that they may have the capacity for same-sex sexuality. Gender did not significantly moderate the effect of the continuous manipulation on sexual orientation change contrary to Hypotheses 2A. Moreover, supporting Hypothesis 1, reading the continuous account caused all participants, regardless of political orientation, to report less certainty about their sexual orientation. Given that college students may be particularly open to sexual identity exploration it was necessary to examine these findings in a heterogeneously-aged community sample. Study 2 failed to directly replicate Study 1 in a nation-wide, more heterogeneously-aged, community sample. Nevertheless, manipulating sexual orientation impacted sexual orientation self-ratings in line with Hypothesis 1. Whereas only 8% of participants in the control condition reported being non-exclusively heterosexual post manipulation, 36% of those who read the continuous account, and 21% who read the fluid account subsequently described themselves as non-exclusively heterosexual post manipulation in this between-subject designed study. These shifts occurred irrespective of political orientation, or gender (contrary to Hypotheses 2A and 2B). Furthermore, in Study 2, the continuous account not only led heterosexuals to report less certainty about their sexual orientation, but also to express greater willingness to engage in sexual or romantic interactions with members of the same-sex. This latter finding may have been significant in Study 2 but not in Study 1 given the substantial difference in power, with the Study 2 sample size offering greater power to find a significant effect for between-subject variables, such as those.

As mentioned, unlike in Study 1, in Study 2 manipulations influenced sexual orientation self-ratings irrespective of the political orientation of participants (contrary to Hypothesis 2B). It is possible that the effects of the continuous condition were stronger in Study 2 (national sample; average age 45) than Study 1 (college sample; average age 19) because these notions of sexual orientation were more novel for the national sample who were on average middle-aged and more politically conservative (i.e., a ceiling effect may have reduced the strength of shifts in the college sample—who were perhaps already well accustomed to continuous notions of sexuality due to young age and political progressivism). Another possibility is that the differences in the design of the studies contributed to the differences in the effect of political orientation. In the first study, a within-subject assessment allowed us to assess actual within-subject changes following the manipulation. As such, it meant that participants were willing to show changes in their self-perceptions following the manipulation. As politically conservative students view same-sex attraction more negatively than their progressive counterparts^35^, it was arguably less taxing for the progressive (and moderates) to admit that the information in the manipulation (legitimizing gradations of heterosexuality) led them to explicitly recognise some low level of same-sex attraction in themselves. For conservatives, on the other hand, such an admission may have been more taxing and thus, less frequent, leading to the observed differences. In Study 2, on the other hand, no pre-manipulation indication of their Kinsey-type identity label was reported, thus the willingness to show that they accepted the information in the manipulation by changing their identity label immediately following the manipulation was absent, allowing the participants to avoid showing explicit endorsement of the information about continuity (or fluidity) of sexual orientation. Thus, the nature of the studies’ designs may have led conservative individuals to respond differently to the manipulations.

Across studies, neither the discrete nor stable manipulations demonstrated effects on sexual orientation self-concept—and specifically, contrary to Hypothesis 3—they failed to increase exclusivity of heterosexual feelings or decrease sexual orientation uncertainty or willingness to engage in same-sex encounters. This may be interpreted to suggest that discrete and stable notions of sexual orientation were perhaps the default assumptions in our sample. Furthermore, in Study 2, increased rates of non-exclusive heterosexual identification were observed also among those in the discrete condition (although this was a non-significant trend). On reflection, it is possible that the discrete condition provided scientific evidence that bisexuality exists—which is counter to the binary view of sexual orientation that is still predominant in society—particularly in older generations^6^ and those who are more politically conservative^36^. This may have resulted in more self-reports in the bisexual spectrum in some participants. However, the failure to observe this trend in Study 1 may indicate that this finding is spurious and requires future replication.

Contrary to our Hypotheses 2A, women appeared no more likely than men to report non-exclusive heterosexuality following exposure to informational accounts. At face value this is surprising given considerable evidence the female sexual orientation is more malleable than male sexual orientation^21,22,37^, and given that heterosexual men are likely to experience greater internal resistance^28^, and greater social backlash^24,30^, to adopting a non-exclusive heterosexual orientation. However, a closer look at Study 1 may explain why we did not observe gender differences in these effects. Pre-manipulation, men were much more likely than women to report an exclusively heterosexual orientation (91.7% versus 68.3%), and as discussed above, we observed a trend wherein those who were exclusively heterosexual at pre-test demonstrated stronger shifts following exposure to the continuous manipulation than those who were non-exclusively heterosexual at pre-test. Thus, a ceiling effect among the subset of women who already rated themselves as non-exclusively heterosexual at pre-test, may explain why women did not demonstrate stronger increases in non-exclusive heterosexuality than men.

Providing convergent evidence that the continuous account of sexual orientation can influence how heterosexuals view their sexual orientation—participants who read the continuous account were more uncertain of their sexual orientation than those in the control group. This may prove to be a temporary phase as individuals try to make sense of their sexual orientation in light of the new information, or alternatively, it may be a more permanent phenomenon which occurs as individuals move away from categorical and binary understandings of sexual orientation^2,38^. Moreover, in Study 2, reading the continuous account of sexual orientation increased participant’s willingness to engage in sexual/romantic interactions with members of the same-sex, relative to controls. Whether these behavioral intentions manifest in actual same-sex encounters will require future research. Critically, although those who read continuous and fluid accounts of sexual orientation were more likely to report a non-exclusive heterosexual orientation than controls, it is difficult to assess the generalizability of these effects to a real-world context, including whether the effects of reading these accounts persists over time (i.e., did our brief intervention permanently affect how participants view their sexual orientation?). Longitudinal studies are required to assess the stability of these effects on self-perceived sexual orientation—and thus these findings should be interpreted with caution. Such studies will benefit from integrating elements that can shed light on the mechanisms that are involved in the transitions away from the completely heterosexual descriptors. As perceptions of homogeneity are considered to be central to essentializing social categories based on elements such as sexual orientation^39^, and this essentialist tendency plays a role in making the members of such groups (e.g., heterosexual vs non-heterosexual individuals) seem more distinct from each others^40^, the role of perceptions of continuity and fluidity in reducing essentialism-derived taxometric notions offers a promising direction to explore.

Our manipulation of sexual orientation focused on influencing people’s perception of the continuity/discreteness/fluidity/stability of sexual arousal/sexual attraction, which although at the heart of what most people mean by sexual orientation^41^, is not the only thing which informs people’s sexual orientation. For instance, had our manipulations discussed continuity or fluidity in romantic feelings or pair bonding feelings—these may have impacted sexual orientation self-ratings in different ways. Arguably, as romantic feelings may be less gender specific than sexual arousal/attraction^42^, at least in males, including romantic themes (e.g., evidence that feelings of emotional intimacy/crushes toward members of the same-sex indicate non-exclusive heterosexuality) in our manipulation may have resulted in larger shifts in self-ratings. Future research may examine this possibility.

An intriguing possibility is that heterosexuals who hold a continuous or fluid view of sexual orientation are less prejudiced toward gay, lesbian, or bisexual peoples. Such a prediction is supported by evidence that indicates that perceiving sexual orientation as discrete is associated with greater anti-gay prejudice^8^. On the other hand, a recent study found that when heterosexual men are exposed to information that blurs the distinction between themselves and homosexual men, they enact greater homophobia, to re-establish their distinctiveness^28^. Future research clarifying how continuous and fluid notions of sexual orientation impact sexual prejudice is therefore of the upmost importance. Further, although the present research focused solely on heterosexual populations, future research may also examine how exposure to continuous and fluid notions of sexual orientation influences how gay men and lesbian women conceive of their sexual orientation. The potential effects of such exposure on shifts in reported sexual orientation and on levels of internalized homophobia are valuable, needed explorations.

Our findings also bring into question the meaning of “mostly” (e.g., “mostly same-sex attracted”, mostly opposite-sex attracted”) ratings on Kinsey-type measures. As commonly interpreted by researchers and lay people alike, individuals who report different positions on a Kinsey-type scale are thought to possess different sexual orientations^43^. But how then do we make sense of the present findings—in which participants’ self-ratings changed following our manipulations? Did we change the sexual orientation of our participants? Surely not. To make sense of the shifts observed we need to recognize that measures such as the Kinsey scale can only possibly assess “self-perceived sexual orientation”^44^. Although self-perceived sexual orientation is partly informed by actual sexual/romantic experiences (which gender/s we find sexually arousing, crush on, fantasize about, have sex with) these experiences are filtered through appraisals of these thoughts, feelings, and behaviours based on a range of personal beliefs and attitudes. This means that two individuals, with identical sexual experiences, could report quite different sexual orientations. The present study found that manipulating participants beliefs about sexual orientation changed how they *interpreted* their sexual/romantic experiences and the subsequent global assessment they made when rating their sexual orientation. As considerable effort has been undertaken to understand mental health^45,46^, substance use^47,48^, sexual health^49–51^, discrimination^52^, and even physiological differences^43,53–57^ between exclusive and non-exclusive heterosexual individuals, clarifying the cognitive and attitudinal variables that may predispose a heterosexual person to adopt respective labels is surely important if we are interested in the causes of differences (e.g., mental health, sexual health) between exclusive heterosexual and non-exclusive heterosexual populations.

Our findings suggest that non-exclusive heterosexual orientations might become more prevalent as continuous and fluid notions of sexuality become more culturally mainstream and provide currently-identified heterosexuals with more nuanced ways of describing themselves. We should stress that present findings do not support the contention that sexual orientation (the underlying compass that directs our sexual/romantic feelings) can be changed. Rather we show that how people understand and label their experiences can influenced by exposure to certain theories of sexual orientation, which arguably more accurately reflect their underlying feelings.

## Supplementary Information

Supplementary Information.

## Data Availability

All data is available in the main text or the Supplementary Information.

## References

[1] Morandini JS, Blaszczynski A, Ross MW, Costa DS, Dar-Nimrod I (2015). Essentialist beliefs, sexual identity uncertainty, internalized homonegativity and psychological wellbeing in gay men. J. Couns. Psychol..

[2] Morandini J, Blaszczynski A, Costa DS, Godwin A, Dar-Nimrod I (2017). Born this way: Sexual orientation beliefs and their correlates in lesbian and bisexual women. J. Couns. Psychol..

[3] Arseneau JR, Grzanka PR, Miles JR, Fassinger RE (2013). Development and initial validation of the Sexual Orientation Beliefs Scale (SOBS). J. Couns. Psychol..

[4] Daniel, V. *Gender-neutral, ­sexually** fluid pop star Miley Cyrus married actor Liam Hemsworth*. https://www.lgbtqnation.com/2018/12/gender-neutral-%C2%ADsexually-fluid-pop-star-miley-cyrus-married-actor-liam-hemsworth/ (Accessed 3 April 2019) (2018).

[5] Savin-Williams, R. C. *Why ‘Mostly Straight’ Men Are a Distinct Sexual Identity*. https://time.com/5026092/mostly-straight-sexual-identity-bisexual-gay/ (Accessed 14 March 2019) (2017).

[6] Ballard, J. *More young Americans now identify as bisexual*. https://today.yougov.com/topics/relationships/articles-reports/2018/06/18/more-young-americans-now-identify-bisexual (Accessed 15 April 2019) (2018).

[7] Kinsey AC, Pomeroy WB, Martin CE (1948). Sexual Behavior in the Human Male.

[8] Haslam N, Levy SR (2006). Essentialist beliefs about homosexuality: Structure and implications for prejudice. Pers. Soc. Psychol. Bull..

[9] Whitley BE (1990). The relationship of heterosexuals' attributions for the causes of homosexuality to attitudes toward lesbians and gay men. Pers. Soc. Psychol. Bull..

[10] Hegarty P (2002). ‘It's not a choice, it's the way we're built’: Symbolic beliefs about sexual orientation in the US and Britain. J. Community Appl. Soc. Psychol..

[11] Jayaratne TE (2006). White Americans' genetic lay theories of race differences and sexual orientation: Their relationship with prejudice toward Blacks, and gay men and lesbians. Group Process. Intergroup Relat..

[12] Israel T, Mohr JJ (2004). Attitudes toward bisexual women and men: Current research, future directions. J. Bisexuality.

[13] Morandini JS, Blaszczynski A, Dar-Nimrod I (2016). Who adopts queer and pansexual sexual identities?. J. Sex Res..

[14] Dahlgreen, W. & Shakespeare, A. E. 1 in 2 young people say they are not 100% heterosexual. *YouGov,* https://yougov.co.uk/news/2015/08/16/half-young-not-heterosexual (Accessed 2 April 2019) (2015).

[15] Savin-Williams RC, Vrangalova Z (2013). Mostly heterosexual as a distinct sexual orientation group: A systematic review of the empirical evidence. Dev. Rev..

[16] Saad, L. *U.S. acceptance of gay/lesbian relations is the new normal*. http://www.gallup.com/poll/154634/Acceptance-Gay-Lesbian-Relations-New-Normal.aspx (Accessed 13 March 2019) (2012).

[17] Chaiken S, Zanna MP, Olsen JM, Herman CP (1987). The heuristic model of persuasion. Social influence: The Ontario symposium.

[18] Cacioppo JT, Petty RE (1984). The elaboration likelihood model of persuasion. ACR N. Am. Adv..

[19] Petty RE, Cacioppo JT, Goldman R (1981). Personal involvement as a determinant of argument-based persuasion. J. Pers. Soc. Psychol..

[20] Klucharev V, Smidts A, Fernández G (2008). Brain mechanisms of persuasion: How ‘expert power’ modulates memory and attitudes. Soc. Cogn. Affect. Neurosci..

[21] Chivers ML, Rieger G, Latty E, Bailey JM (2004). A sex difference in the specificity of sexual arousal. Psychol. Sci..

[22] Baumeister RF (2000). Gender differences in erotic plasticity: The female sex drive as socially flexible and responsive. Psychol. Bull..

[23] Vrangalova Z, Savin-Williams RC (2012). Mostly heterosexual and mostly gay/lesbian: Evidence for new sexual orientation identities. Arch. Sex. Behav..

[24] Herek GM (2002). Gender gaps in public opinion about lesbians and gay men. Public Opin. Q..

[25] Herek GM (2009). Hate crimes and stigma-related experiences among sexual minority adults in the United States: Prevalence estimates from a national probability sample. J. Interpers. Violence.

[26] Vandello JA, Bosson JK, Cohen D, Burnaford RM, Weaver JR (2008). Precarious manhood. J. Pers. Soc. Psychol..

[27] Vandello JA, Bosson JK (2013). Hard won and easily lost: A review and synthesis of theory and research on precarious manhood. Psychol. Men Masc..

[28] Vieira de Figueiredo C, Pereira CR (2021). The effect of gender and male distinctiveness threat on prejudice against homosexuals. J. Pers. Soc. Psychol.

[29] Jost JT, Glaser J, Kruglanski AW, Sulloway FJ (2003). Political conservatism as motivated social cognition. Psychol. Bull..

[30] Herek GM (2002). Heterosexuals' attitudes toward bisexual men and women in the United States. J. Sex. Res..

[31] Savin-Williams RC (2014). An exploratory study of the categorical versus spectrum nature of sexual orientation. J. Sex Res..

[32] Mohr JJ, Kendra MS (2011). Revision and extension of a multidimensional measure of sexual minority identity: The Lesbian, Gay, and Bisexual Identity Scale. J. Couns. Psychol..

[33] Hayes, A. F. PROCESS: A versatile computational tool for observed variable mediation, moderation, and conditional process modeling. http://www.afhayes.com/public/process2012.pdf (2012).

[34] ABS. Australian demographic statistics, Mar 2017. Cat No. 3101.0. Canberra: ABS (2017).

[35] Lottes IL, Kuriloff PJ (1992). The effects of gender, race, religion, and political orientation on the sex role attitudes of college freshmen. Adolescence.

[36] Feinstein BA, Dyar C, Bhatia V, Latack JA, Davila J (2015). Conservative beliefs, attitudes toward bisexuality, and willingness to engage in romantic and sexual activities with a bisexual partner. Arch. Sex. Behav..

[37] Diamond LM (2008). Sexual Fluidity: Understanding Women’s Love and Desire.

[38] Diamond LM, Omoto AM, Kurtzman HS (2006). What we got wrong about sexual identity development: Unexpected findings from a longitudinal study of young women. Sexual Orientation and Mental Health: Examining Identity and Development in Lesbian, Gay, and Bisexual People.

[39] Dar-Nimrod I, Heine SJ (2011). Genetic essentialism: On the deceptive determinism of DNA. Psychol. Bull..

[40] Heine SJ, Dar-Nimrod I, Cheung BY, Proulx T (2017). Essentially biased: Why people are fatalistic about genes. Adv Exp Soc Psychol.

[41] Bailey JM (2009). What is sexual orientation and do women have one?. Neb. Symp. Motiv. Neb. Symp. Motiv..

[42] Diamond LM (2003). What does sexual orientation orient? A biobehavioral model distinguishing romantic love and sexual desire. Psychol. Rev..

[43] Savin-Williams RC (2017). An exploratory study of exclusively heterosexual, primarily heterosexual, and mostly heterosexual young men. Sexualities.

[44] Preciado MA, Johnson KL, Peplau LA (2013). The impact of cues of stigma and support on self-perceived sexual orientation among heterosexually identified men and women. J. Exp. Soc. Psychol..

[45] Kuyper L, Bos H (2016). Mostly heterosexual and lesbian/gay young adults: Differences in mental health and substance use and the role of minority stress. J. Sex Res..

[46] Maheux AJ (2020). Depressive symptoms among mostly heterosexual adolescents. J. Gay Lesbian Ment. Health..

[47] Hughes T, Szalacha LA, McNair R (2010). Substance abuse and mental health disparities: Comparisons across sexual identity groups in a national sample of young Australian women. Soc. Sci. Med..

[48] Kuyper L, Fokkema T (2011). Minority stress and mental health among Dutch LGBs: Examination of differences between sex and sexual orientation. J. Couns. Psychol..

[49] Lorenz TK (2019). Brief report: Sexual wellbeing in heterosexual, mostly heterosexual, and bisexually attracted men and women. Int. J. Sex. Health.

[50] Corliss HL, Austin SB, Roberts AL, Molnar BE (2009). Sexual risk in “mostly heterosexual” young women: Influence of social support and caregiver mental health. J. Womens Health.

[51] Austin SB, Roberts AL, Corliss HL, Molnar BE (2008). Sexual violence victimization history and sexual risk indicators in a community-based urban cohort of “mostly heterosexual” and heterosexual young women. Am. J. Public Health.

[52] Talley AE, Grimaldo G, Wilsnack SC, Hughes TL, Kristjanson AF (2016). Childhood victimization, internalizing symptoms, and substance use among women who identify as mostly heterosexual. LGBT Health.

[53] Savin-Williams RC, Rieger G, Rosenthal AM (2013). Physiological evidence for a mostly heterosexual orientation among men. Arch. Sex. Behav..

[54] Dawson SJ, Fretz KM, Chivers ML (2017). Visual attention patterns of women with androphilic and gynephilic sexual attractions. Arch. Sex. Behav..

[55] Chivers ML, Bouchard KN, Timmers AD (2015). Straight but not narrow; Within-gender variation in the gender-specificity of women’s sexual response. PLoS One.

[56] Suschinsky KD, Dawson SJ, Chivers ML (2017). Assessing the relationship between sexual concordance, sexual attractions, and sexual identity in women. Arch. Sex. Behav..

[57] Morandini JS, Veldre A, Holcombe AO, Hsu K, Lykins A, Bailey JM, Dar-Nimrod I (2019). Visual attention to sexual stimuli in mostly heterosexuals. Arch. Sex. Behav.

