# Supplementary material for: Exposure to continuous or fluid theories of sexual orientation leads some heterosexuals to embrace less-exclusive heterosexual orientations
Source: Sci Rep. 2021 Aug 16;11:16546. doi: 10.1038/s41598-021-94479-9 (PMC8368213; doi:10.1038/s41598-021-94479-9)
Supplement: Supplementary file 1 — Supplementary Information. [file 41598_2021_94479_MOESM1_ESM.docx]

Supplementary Materials for

Exposure to continuous or fluid theories of sexual orientation leads some heterosexuals to embrace less-exclusive heterosexual orientations

James S. Morandini^1^, Liam DaCosta^1^ & Ilan Dar-Nimrod^1,2^

^1^The School of Psychology, University of Sydney, Australia ^2^The Charles Perkins Centre, University of Sydney, Australia Correspondence to: [james.morandini@sydney.edu.au](mailto:james.morandini@sydney.edu.au)

# This PDF file includes:

Materials and Methods Supplementary Text

# Materials and Methods

In addition to variables examined in this manuscript (i.e., those related to perception of one’s own sexual orientation) the experiment also included scale measures of attitudes toward bisexuals^1^, homosexuals^2^, and beliefs about the nature and etiology of sexual orientation^3^ which were completed following manipulation. These additional variables were not relevant to present studies focus and so were not examined in this manuscript.

Below is additional information on methodology and materials for Study 1 and Study 2.

Study 1

**Participants:** Participants were recruited from a first-year psychology participation pool at The University of Sydney. To be eligible for participation, students were required to speak fluent English, identify as heterosexuals (“straight”), and identify with the gender they were assigned at birth in the pre-screen survey. Only students who had previously rated (in mass testing for the entire subject pool) their sexual orientation as “straight” were able to see the recruitment advert on SONA (the psychology research participation platform used at our institution).

Participants were excluded from analyses if they completed more than one out of five questions incorrectly on a manipulation check, took excessively long to complete the study, or failed to provide a value on key dependent variables. Of the 240 participants who completed the study, 42 were excluded for failing the manipulation check, 14 for taking over an hour to complete the study, and four for incomplete responses. After applying the exclusion criteria, 45 participants (33 female) were in the continuous condition, 67 (46 female) in the discrete condition, and 58 (41 female) in the control. The final sample consisted of 180 participants (120 female; 60 male) who ranged in age from 18 to 53 (M =

19.51 years; SD = 3.76).

**Procedure**: The study was completed online. After providing informed consent, participants completed a demographics survey, including a pre-test measure of their sexual orientation. Then, participants were randomly assigned to one of three conditions: continuous, discrete, or control. In the continuous condition, participants read a one-page article detailing recent evidence that sexual orientation exists on a continuum inclusive of mostly heterosexual orientations. In the discrete condition, the article presented evidence that sexual orientation exists in three discrete forms, heterosexuality, bisexuality, and homosexuality. The third condition (control condition) was a popular science article on the effects of global warning.

After reading the article, participants were asked five true or false questions based on the information they had just read (manipulation check). Participants then re-rated their sexual orientation (on the same pre-test measure); they also responded to questions assessing their certainty about their sexual orientation and their willingness to engage in same-sex sexual or romantic behaviours. Following manipulation, participants also completed a range of other measures, which were not relevant to this study. These were the Sexual Orientation Beliefs Scale^3^, Attitudes Regarding Bisexuals Scale^1^, the Modern Homophobia Scale^2^, single item warmth thermometers and single items assessing perceived legitimacy of gay men, lesbian women, bisexual men, and bisexual women, and attractiveness ratings of 10 models of each gender.

**Experimental manipulation**. There were three conditions (continuous, discrete, and control). The wordings used in the continuous and discrete conditions were closely matched— with both using the same template — but with specific aspects of sentences changed to reflect either sexual orientation as existing on a continuum or as existing in three discrete categories (heterosexual, bisexual, or homosexual). Participants were instructed “Please read through the following article. Read through it as many times as you like. You will be asked to complete a short comprehension task based on this article once you have read it.”

The **continuous manipulation** read as follows:

“*There is increasing evidence that sexual orientation exists on a spectrum from heterosexual to homosexual, with many people falling somewhere in-between (e.g., mostly heterosexual, bi-leaning heterosexual, bisexual, bi-leaning homosexual, mostly homosexual). Although traditionally sexual orientation was thought to exist in only two forms (heterosexuality and homosexuality), Professor Ritch Savin-Williams and Dr Gerulf Rieger from Cornell University have provided amongst the strongest evidence to date that sexual orientation does appear to be a spectrum.*

*Researchers have found that, when given the option, a significant minority of individuals report that their sexual orientation falls somewhere in-between exclusively heterosexual and exclusively homosexual, such as mostly heterosexual (i.e., mostly sexually attracted to the opposite-sex but occasionally attracted to the same-sex), bisexual (i.e., sexually attracted to both the same- and opposite- sex), or mostly homosexual (i.e., mostly sexually attracted to the same-sex but occasionally attracted to the opposite-sex). Indeed, in their review of nationally representative survey data, Savin-Williams found that mostly heterosexuals were the largest group of same-sex attracted individuals, comprising around 10-20% of the public, which is a significantly larger proportion than the ~3% that reported being homosexual.*

*
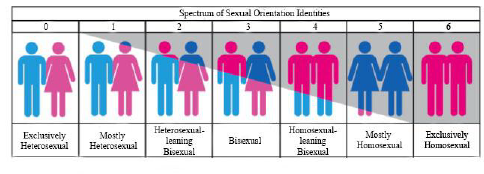
*

*Historically there has been some contention over the existence of the sexual orientation spectrum, with some arguing that those who report being mostly heterosexual, bisexual, or mostly homosexual are actually homosexual individuals who haven’t fully “come out”, or heterosexual individuals who are just experimenting with their sexuality. Although this may be true of a small number of individuals, recent studies find that most individuals who report being at intermediate points on the sexual orientation spectrum report matching patterns of sexual attraction and sexual behaviour. For instance, mostly heterosexuals are more likely to engage in same-sex sexual activity than those who report being exclusively heterosexual, but are less likely to engage in same-sex activity than bisexuals. Moreover, some studies have found that mostly heterosexual, bisexual, and mostly homosexual attractions are just as stable as heterosexuality or homosexuality, with little evidence that they are a temporary phase.*

*Recently, Savin-Williams and Rieger attempted to demonstrate the existence of a sexual orientation spectrum using objective measures of sexual orientation (given that people’s self-reported attractions may be unreliable). Assessing sexual orientation objectively involves measuring genital bloodflow or pupil dilation in the lab whilst people are shown erotic films of men and women separately. Past studies have found that genital arousal and pupil dilation patterns are strongly related to one’s sexual orientation – heterosexual people experience genital arousal/pupil dilation when viewing attractive members of opposite-sex, and homosexuals experience genital arousal/pupil dilation when viewing attractive members of the same-sex. As such, if sexual orientation is a spectrum, it would be expected that those reporting mostly heterosexual, bisexual, or mostly homosexual orientations should show corresponding patterns of sexual arousal when tested in the lab.*

*The researchers recruited men and women from across the sexual orientation continuum (i.e., exclusively heterosexual, mostly heterosexual, bi-leaning heterosexual, bisexual, bi-leaning homosexual, mostly homosexual, and exclusively homosexual orientations) and examined their genital arousal whilst they viewed pornography depicting members of the same- and opposite- sex. In line with their self-reported attractions, physiological sexual arousal was also on a spectrum. For instance, mostly heterosexuals showed slightly more genital arousal to same-sex stimuli than did exclusively heterosexual individuals, but less genital arousal than individuals who were substantially bisexual.*

*The overwhelming evidence at present supports the idea that sexual orientation does exist on a spectrum from heterosexual through to homosexual, with many people falling somewhere in-between.*”

Continuous Manipulation Check (true or false):

1. A significant minority of individuals report that their sexual orientation falls somewhere in-between exclusively heterosexual and exclusively homosexual
2. Mostly heterosexuals are not the largest group of same-sex attracted individuals
3. Around 10-20% of the general public identify as mostly heterosexual
4. Mostly heterosexuals showed discrete condition slightly more genital arousal to same-sex stimuli than did exclusively heterosexual individuals
5. Evidence at present supports the idea that sexual orientation does not exist on a spectrum from heterosexual through to homosexual”

The **discrete manipulation** read as follows:

*“There is increasing evidence that sexual orientation exists in three discrete categories:*

*heterosexual, bisexual, and homosexual. Although traditionally sexual orientation was*

*thought to exist in only two forms (heterosexuality and homosexuality), Professor Ritch*

*Savin-Williams and Dr Gerulf Rieger from Cornell University have provided amongst the*

*strongest evidence to date that bisexuality is a stable “third” type of sexual orientation.*

*Researchers have found that, when given the option, a significant minority of*

*individuals report that their sexual orientation is not heterosexual (only sexually attracted to*

*the opposite-sex), or homosexual (only sexually attracted to the same-sex), but that they’re*

*instead bisexual (sexually attracted to both the same- and opposite-sex). Indeed, in their*

*review of nationally representative data, Savin-Williams found that bisexuals were the largest*

*group of same-sex attracted individuals, comprising around 10-20% of the general public,*

*which is a significantly larger proportion than the 3% that report being homosexual.*

*Historically there has been some contention over the existence of bisexuality, with*

*some arguing that those who report being bisexual are actually homosexual individuals who*

*haven’t fully “come out”, or heterosexual individuals who are just experimenting with their*

*sexuality. Although this may be true of a small number of individuals, recent studies find that*

*most bisexuals report bisexual patterns of sexual attraction and sexual behaviour. For*

*instance, bisexuals are more likely to engage in same-sex sexual activity than those who*

*report being heterosexual, but are less like to engage in same-sex activity than homosexuals.*

*Moreover, some studies of bisexual individuals have found that bisexuality is just as stable as*

*heterosexuality or homosexuality, with little evidence that it is a temporary phase.*

*Recently, Savin-Williams and Rieger attempted to demonstrate the existence of*

*bisexuality using objective measures of sexual orientation (given that people’s self-reported*

*attractions may be unreliable). Assessing sexual orientation objectively involves measuring*

*genital bloodflow or pupil dilation in the lab whilst people are shown erotic films of men and*

*women separately. Past studies have found that genital arousal and pupil dilation patterns are*

*strongly related to one’s sexual orientation – heterosexual people experience genital*

*arousal/pupil dilation when viewing attractive members of opposite-sex, and homosexuals*

*experience genital arousal/pupil dilation when viewing attractive members of the same-sex.*

*As such, if bisexuality is a distinct sexual orientation, it would be expected that those*

*reporting bisexual orientations would show substantial sexual arousal to* ***both*** *men and*

*women when tested in the lab.*

*The researchers recruited men and women from who identified as bisexual, as well as*

*homosexual and heterosexual individuals, and examined their genital arousal whilst they*

*viewed pornography depicting members of the same- and opposite- sex. In line with their*

*self-reported attractions, bisexuals demonstrated a distinct pattern of sexual arousal compared*

*to heterosexual and homosexuals. That is, bisexuals demonstrated substantial genital arousal*

*to both men and women, whereas homosexuals were only genitally aroused to same-sex*

*stimuli, and heterosexuals were only genitally aroused by opposite- sex stimuli.*

*The overwhelming evidence at present supports the idea that sexual orientation does*

*exist in three discrete categories, heterosexual vs. homosexual vs. bisexual.”*

Discrete Manipulation Check (true or false)

1. Based on nationally representative data, bisexuals are ***not*** the largest group of same-sex attracted individuals
2. Some studies of bisexual individuals have found that bisexuality is just as stable as

heterosexuality or homosexuality

1. Past studies have found that genital arousal and pupil dilation patterns are ***not*** strongly related to one’s sexual orientation
2. Bisexuals demonstrated substantial genital arousal to both men and women
3. Evidence at present supports the idea that sexual orientation does exist in three discrete categories, heterosexual vs. homosexual vs. bisexual

The **control condition** was not matched for content (it comprised a popular science article on the effects of climate change in the high artic) but was matched for length. It read as follows:

“*The evidence comes from those silent witnesses, the natural things that respond to climate signals. The relatively new science of phenology – the calendar record of first bud,*

*first flower, first nesting behaviour and first migrant arrivals – has over the last three decades repeatedly confirmed meteorological fears of global warming as a consequence of the combustion of fossil fuels.*

*Researchers say the evidence from the plant world is consistent with the instrumental*

*record: 2016 was the hottest year ever recorded, and it was the third record-breaking year in*

*succession. Sixteen of the hottest years ever recorded have happened in the 21st century.*

*The most dramatic changes are observed in the high Arctic, the fastest-warming place*

*on the planet, according to a study in Biology Letters. As the polar sea ice retreats, the*

*growing season gets ever longer and arrives earlier.*

*The pattern is not consistent: grey willow sticks to its original timetable, and dwarf birch*

*growth has advanced about five days earlier for each decade. But the sedge, almost four*

*weeks ahead of its timetable in a decade, holds the record, according to a study that observed*

*one plot at a field site in west Greenland, 150 miles inland, for 12 years.*

*“When we started studying this, I never would have imagined we’d be talking about a*

*26-day per decade rate of advance,” says Eric Post, a polar ecologist at the University of*

*California, Davis, department of wildlife, fish and conservation biology, who has been*

*studying the Arctic for 27 years. “That’s almost an entire growing season. That’s an eye opening rate of change.”*

*Caribou come to the study site during the calving season, to graze on the rich plant*

*life of the brief Arctic summer. The caribou set their migration calendar by day-length. But*

*some of the plants prefer to respond to temperature, which means that by the time the caribou*

*arrive, the plants have flourished and the pickings are not as nutritious. So fewer calves are*

*born and more die.*

*“That’s one example of the consequences of this for consumer species like caribou,*

*who have a limited window to build up resources before going into the next winter,” Post*

*says. “With the most recent study, we’re taking a step towards understanding how extensive*

*and cryptic the effects of sea ice loss might be in the Arctic.”*

*Further south, spring keeps on springing, according to the US Geological Survey*

*(USGS), which has just published a new set of maps based on phenological observations.*

*And, once again, an early spring doesn’t mean a sunnier, kinder world for everybody.*

*Ticks and mosquitoes become more active, pollen seasons last longer. Crops could flourish or be at risk from a sudden late frost or summer drought.*

*Plants could bloom before the arrival of the birds, bees and butterflies that feed on and*

*pollinate the flowers, with consequences for both the plant and the pollinator.*

*“While these earlier springs might not seem like a big deal – and who among us*

*doesn’t appreciate a balmy day or a break in dreary winter weather – they pose significant*

*challenges for planning and managing important issues that affect our economy and our*

*society,” says one of the authors of the report, Dr Jake Weltzin, a USGS ecologist and national director of the USA National Phenology Network.*”

Control Manipulation Check (true or false):

1. 2016 was not the hottest year ever recorded.
2. The most dramatic changes due to global warming are observed in the high Arctic.
3. Sixteen of the hottest years ever recorded have happened in the 21st century.
4. Caribou come to the study site during the calving season, to graze on the rich plant life of the brief Arctic summer.
5. Global warming is not a consequence of the combustion of fossil fuels.

# Data Preparation

The conditions were dummy coded into two variables; the first contrasted continuous and control and the second contrasted discrete and control. Changes in sexual orientation were operationalised as the difference in self-rating (on the 9-point scale) before and after the manipulation. A positive difference-score represented a shift toward more same-sex attraction whereas a negative difference-score represented a shift toward less same-sex attraction. The responses to the sexual orientation uncertainty and willingness scales were extremely non- normal, which is expected given the heterosexual sample. Whereas dichotomization of continuous variables typically results in a significant loss in power, one of the circumstances in which dichotomization is acceptable, is when the responses are extremely skewed. Subsequently, responses were dichotomized into two groups for both variables: those who were not at all uncertain (“strongly disagree”), or willing, and those who were less certain (“somewhat disagree” to “strongly agree”), or more willing (“somewhat willing” to “extremely willing”). Where significant interactions were observed, we report post-hoc simple slopes analyses using Hayes’s PROCESS Macro, version 3.0^5^.

# Data Analyses

For each dependent variable (DV: sexual orientation, sexual orientation uncertainty, willingness to engage in same-sex sexual/romantic behaviours), regression models took the same form. In Step 1, we examined the main effect dummy-coded continuous versus control variable, and the dummy-coded discrete versus control variable, on the dependent variable of interest. In Step 2 (which was only interpreted if the R-Square change for Step 2 was significant), we examined the main effect of political orientation (in continuous form), gender (male versus female), and added interactions between political orientation×gender, continuous condition×political orientation, continuous condition×gender, discrete condition×political orientation, and discrete condition×gender. Hierarchical Linear Regressions were used to analyse data for the sexual orientation difference-scores. Binomial Logistic Regressions were used to analyse sexual orientation uncertainty and willingness to engage in sexual/romantic interactions with the same-sex, given the binary nature of these dependent variables.

We also included a secondary analysis (in Supplemental Materials only) in which we compared continuous versus discrete conditions directly on all dependent variables of interest. Otherwise, these analyses mirrored those described (e.g., in Step 1 we examined discrete versus continuous condition, and in Step 2 we examined gender, political orientation, and relevant interactions with the Discrete versus Continuous condition).

To reduce the likelihood of committing Type-I errors, a Bonferroni correction was applied to account for the multiple DVs. Given that we examined three DVs, we used a *p*- value of .0167 (i.e., .05/3) to determine statistical significance.

# Results

**Sexual Orientation Self-Ratings**

Using hierarchical linear regression models discussed above, we tested our main hypotheses, that those who read the continuous account of sexual orientation, would shift their ratings of their own sexual orientation to be less exclusively heterosexual (relative to the control group), unlike those who read the discrete account, who would either not change, or shift toward a more exclusive heterosexual orientation. We failed to find support for these hypotheses; no main effect of either the continuous condition, *t*(170) *=* 1.913, *β =* .166, *p =*.057, or discrete condition, *t*(170) *=* .874, *β =* .076, *p =* .383, were observed. However, political orientation significantly moderated the effect of the exposure to the continuous condition on sexual orientation self-ratings, *t*(170) *=* 3.041, *β =* .276, *p =* .003. Post-hoc simple slopes analyses indicated that only politically progressive individuals demonstrated shifts in sexual orientation self-ratings following exposure to the continuous condition, *t*(176) *=* 3.410, *β =* .462, *p* < .001, CI [.19, .73]. A significant trend finding in the same direction for political moderates was also observed, *t*(176) *=* 2.475, *β =* .241, *p =* .014, CI [.05, .43], however, political conservatives did not shift their self-ratings following exposure to the continuous condition, *t*(176) = .180, *β =* .02, *p* = .857, CI [-.19, .23]. No other interactions were significant, including those examining the interaction of gender and exposure to continuous or discrete conditions (*p*s > .05).

Follow-up analyses assessed whether the shifts in sexual orientation self-ratings observed among progressives, following exposure to the continuous condition, were moderated by whether one rated themselves as exclusively heterosexual or non-exclusively heterosexual at pre-test. That is, did the manipulation shift those who initially rated themselves as exclusively heterosexual to a non-exclusively heterosexual orientation (such as almost exclusively heterosexual) *or* did the manipulation further shift individuals who already rated themselves as non-exclusively heterosexual on the pre-test further toward the homosexual end of the spectrum (e.g., almost exclusively heterosexual individuals shifting to mostly heterosexual). To test this, the following elements were added to the regression model: the main effect of exclusivity at pre-test (0 *=* exclusive heterosexual, 1 *=* non- exclusively heterosexual), two-way interactions between exclusivity×condition (continuous or discrete), and three-way interactions between exclusivity×political orientation×condition (continuous or discrete). The three-way interaction was trending, but non-significant, *t*(165) *=* -2.137, *β =* -.186, *p =* .034. Probing this interaction revealed that the political orientation×continuous condition interaction was significant among individuals who rated themselves as “exclusively heterosexual” at pre-test, *t*(127) *=* 3.216, *β =* .348, *p =* .002, but not among those who rated themselves as “non-exclusively heterosexual” at pre-test, *t*(33) *=* .920, *β =* .281, *p =* .364.

The effect of discreteness on sexual orientation self-ratings was not moderated by the exclusivity of one’s heterosexuality at pre-test, *t*(179) *=* .499, *β =* .060, *p =* .618.

# Secondary Indices

We examined whether effects of manipulations on sexual orientation uncertainty or willingness to engage in same-sex sexual experiences were moderated by whether participants were exclusively or non-exclusively heterosexual at pre-test. No significant effects were observed (*p*s > .05).

*Additional Analysis of Discrete versus Continuous Manipulation*

Using hierarchical linear regression models, we undertook follow-up analysis to examine whether those reading the continuous account of sexual orientation would shift their ratings of their sexual orientation relative to participants who read the discrete account. We failed to find support for this notion with no main effect of discrete versus continuous condition emerging, *t*(121) *=* .966, *β =* .088, *p =*.336. However, as in the main analysis, political orientation significantly moderated the effect of the exposure to the discrete versus continuous condition on sexual orientation self-ratings, *t*(121) *=* 2.782, *β =* .769, *p =* .006. Post-hoc simple slopes analyses indicated that politically progressive individuals demonstrated shifts in sexual orientation self-ratings following exposure to the continuous condition, *t*(121) *= -*2.4017, *β =* .424, *p* = .017, CI [-.77, -.07]. A non-significant trend in the same direction for political moderates was observed, *t*(121) *= -*1.6307, *β =* -.21, *p =* .106, CI [-.46, .04], however, political conservatives did not shift their self-ratings following exposure to the continuous condition, *t*(121) = .0724, *β =* .0104, *p* = .9424, CI [-.27, .29] (See Supplementary Figure 1).

*Secondary Indices*

The continuous versus discrete manipulation had no significant main effect (or interactions) on sexual orientation uncertainty or sexual willingness of participants, *ps* > .05 (see Supplementary Table 1).

Study 2

# Participants

A nationally representative sample was recruited via Qualtrics panels. The final sample consisted of 460 (232 female) heterosexual identified participants. Ages ranged from 18-83 (*M* = 45.65, *SD* = 15.74); 78.7% of the sample identified as White, 13.5% identified as Asian, 1.3% identified as Indigenous Australian, and the remaining 6.5%. The demographic break-down of participants with regard to gender, age, and ethnicity is in line with the national values. Regarding political orientation, 31.3% identified as between extremely conservative and fairly conservative, 33.5% as moderate, 35.2% as between fairly progressive and extremely progressive. The entire sample used the label ‘straight’ to describe their sexual orientation.

# Procedure

As with Study 1, participants gave their informed consent online before beginning the study. Similarly, after completing the demographics, participants were randomly assigned to one of the five conditions. The continuous and discrete manipulations presented the same concepts as in Study 1, whereas Study 2 added two additional conditions – one emphasizing how sexual orientation can change over time (fluid condition), and the other highlighting how sexual orientation remains fixed across time (stable condition). The control condition was a popular science article describing the anatomy of trees. Following the manipulation, participants answered three true or false questions based on the information they had just read (manipulation check), and completed the sexuality measures. They were then reimbursed for their participation and debriefed.

# Materials

**Manipulations**. As mentioned above, the continuous manipulation was revised for Study 2, due to a concern that the continuous condition in Study 1 did not prime to concept of the sexual orientation continuum strongly enough. The discreteness manipulation was also revised to match the length and style of the new continuous manipulation. The revised continuous manipulation read:

“*There is increasing evidence that sexual orientation exists on a spectrum from*

*heterosexual to homosexual, with many people falling somewhere in-between. Although*

*traditionally sexual orientation was thought to exist in only two forms (heterosexuality and*

*homosexuality), Professor Ritch Savin-Williams and Dr Gerulf Rieger from Cornell*

*University have provided the strongest evidence to date that sexual orientation does appear to be a spectrum, with many people experiencing some level of attractions to both genders, even if they are much more attracted to one gender than the other.*

*Researchers have found that, when given the option, a significant number of*

*individuals will report that their sexual orientation falls somewhere in-between exclusively*

*heterosexual and exclusively homosexual, even though the strength of their attractions to men and women may differ. For example, someone may be predominantly heterosexual but still experience of a minor degree of same-sex attractions. Others may hold more equal sexual attraction to men and women. This has led some researchers to argue that most people have some level of attraction to both genders, even if they strongly prefer one gender in particular. Historically, there has been some contention over the existence of the sexual orientation spectrum, with some arguing that people who have some attraction to both men and women simply have not fully "come out", or are heterosexual individuals who are just experimenting with their sexual orientation. Although this may be true of a small number of individuals, recent studies find that most individuals who report being at middle points on the sexual report matching patterns of sexual attraction and sexual behaviour.*

*Recently, Savin-Williams and Rieger attempted to demonstrate the existence of a*

*sexual orientation spectrum using objective measures of sexual orientation (given that*

*people's self-reported attractions may be unreliable). Assessing sexual orientation objectively involves measuring genital blood flow or pupil dilation in the lab whilst people are shown erotic films of men and women separately. In this way, researchers can garner objective data about which gender(s) an individual is sexually attracted to. As such, if sexual orientation is a spectrum, it would be expected that, rather than people showing sexual arousal to only men or only women, people's attractions will be scattered across the spectrum (with people demonstrating slightly different ratios of arousal to men vs. women).*

*The researchers recruited men and women who reported attractions from across the*

*sexual orientation continuum (exclusively heterosexual to exclusively homosexual), and*

*examined their genital arousal whilst they viewed pornography depicting members of the*

*same- and opposite-sex. In line with their self-reported attractions, physiological sexual*

*arousal was on a spectrum. That is, many people demonstrated varying degrees of attraction to men and women (from very strong attraction to one gender and very slight to the other, to almost equal attraction to both genders). Notably, even people who identified as exclusively heterosexual or exclusively homosexual tended to demonstrate a small amount of physiological arousal to their non-preferred gender, indicating that most people are attracted to both men and women, even if it's to a varying degree. The overwhelming evidence at present supports the idea that sexual orientation exists on a spectrum from exclusively heterosexual to exclusively homosexual, with many people*

*falling somewhere in-between.*”

**Continuous** Manipulation Check (true or false)

1. Sexual orientation appears to exist along a spectrum.
2. Sexual orientation is NOT best represented as a spectrum.
3. Many people are sexually attracted to both men and women, even if they are more attracted to one gender over the other.

The discrete manipulation read:

“*There is increasing evidence that sexual orientation exists in three discrete categories:*

*heterosexual, bisexual, and homosexual. Although traditionally sexual orientation was*

*thought to exist in only two forms (heterosexuality and homosexuality), Professor Ritch*

*Savin-Williams and Dr Gerulf Rieger from Cornell University have provided amongst the*

*strongest evidence to date that bisexuality is legitimate “third” type of sexual orientation,*

*with bisexual people experiencing genuine attraction to both genders.*

*Researchers have found that, when given the option, a significant minority of*

*individuals report that their sexual orientation is not heterosexual (only sexually attracted to the opposite-sex), or homosexual (only sexually attracted to the same-sex), but that they’re instead bisexual (sexually attracted to both the same- and opposite-sex).*

*Historically there has been some contention over the existence of bisexuality, with*

*some arguing that those who report being bisexual are actually homosexual individuals who haven’t fully “come out”, or heterosexual individuals who are just experimenting with their sexuality. Although this may be true of a small number of individuals, recent studies find that most bisexuals report bisexual patterns of sexual attraction and sexual behaviour. For instance, bisexuals are more likely to engage in same-sex sexual activity than those who report being heterosexual, but are less like to engage in same-sex activity than homosexuals.*

*Recently, Savin-Williams and Rieger attempted to demonstrate the existence of*

*bisexuality using objective measures of sexual orientation (given that people’s self-reported attractions may be unreliable). Assessing sexual orientation objectively involves measuring genital bloodflow or pupil dilation in the lab whilst people are shown erotic films of men and women separately. In this way, researchers can garner objective data about which gender/s an individual is sexually attracted to. As such, if bisexuality is a legitimate sexual orientation, it would be expected that rather than bisexual people showing sexual arousal to only men or only women, bisexual people will demonstrate substantial sexual arousal to both men and women.*

*The researchers recruited men and women who identified as bisexual, as well as*

*homosexual and heterosexual individuals, and examined their genital arousal whilst they*

*viewed pornography depicting members of the same- and opposite- sex. In line with their*

*self-reported attractions, bisexuals demonstrated a distinct pattern of sexual arousal compared to heterosexual and homosexuals. That is, bisexuals demonstrated substantial genital arousal to both men and women, whereas homosexuals were only genitally aroused to same-sex stimuli, and heterosexuals were only genitally aroused by opposite- sex stimuli. Notably, people who identified as heterosexual demonstrated no measurable attraction to films depicting the same-sex and people identified as homosexual demonstrated no measurable attraction to films depicting the opposite-sex, indicating that only bisexual people are genuinely attracted to both men and women.*

*The overwhelming evidence at present supports the idea that sexual orientation does*

*exist in three discrete categories, heterosexual vs. homosexual vs. bisexual.*”

**Discrete** Manipulation Check (true or false)

1. Sexual orientation appears to exist in discrete categories.
2. Sexual orientation is NOT best represented as categories.
3. Bisexual people are the only sexual orientation attracted to BOTH men and women.

The fluid and stable conditions were matched with each other, but not with the continuous and discreteness condition (which, as in Study 1, were matched with one another). The fluidity condition defined the concept of sexual fluidity and summarized scientific evidence purporting to demonstrate that sexual orientation may shift across the life-course, sometimes multiple times (making it explicit that such shifts are typically outside of individual conscious control and should not be confused with choice). The stability condition defined the concept of sexual stability and present evidence that sexual orientation is fixed once it emerges early in life. Conceptually, matching all four manipulations (continuous, discrete, fluid, stable) was not possible as the ideas presented in the fluid and stable conditions did not naturally reflect the ideas of sexual orientation as categorical or continuous. The fluidity manipulation read as follows:

“*Traditionally, an individual's sexual orientation has been thought to be stable and*

*unchanging overtime. However, over the past several decades, researchers have discovered that some people report unexpected changes in their relative attraction to men versus women across their lives. This capacity for change has been labelled "sexual fluidity."*

*For instance, a woman may experience exclusive sexual attraction to men in*

*adolescence and early adulthood, but then develop attractions to women later on in life,*

*potentially after meeting a particular woman. Importantly, the existence of sexual fluidity*

*does not mean that individuals simply choose their sexual orientation. Typically, shifts in*

*one's attractions are unexpected and outside one's conscious control.*

*It is also important to clarify that these shifts do not represent an individual coming to*

*terms with repressed same-sex desires. Most individuals who are sexually fluid report having had genuine sexual feelings toward both genders across time, and many have maintained successful sexual and romantic relationships with both men and women over time. Moreover, some individuals may become aware of same-sex attractions after a history of genuine heterosexual attraction. Others report the reverse.*

*Lisa Diamond's longitudinal research (where the same people are interviewed*

*multiple times across a number of years) into female sexuality provides the most dramatic*

*demonstration of sexual fluidity. Diamond found that, over time, many women reported shifts in their relative attraction to men versus women, in the gender of their sexual and romantic partners, and in their sexual identity (e.g. changing from describing themselves as lesbian to bisexual, and vice-versa). By way of example, one woman initially reported herself as 100% attracted to women, then 90%, 70%, and 50% at the next three follow up interviews.*

*Although it was initially thought that sexual fluidity may be a relatively rare*

*occurrence, a recent study by Dr Sabra Katz-Wise discovered that many people experience*

*genuine changes in their sexual orientation in their lifetime. Others still have argued that*

*fluidity is a general property of human sexuality - even if it goes unexpressed in many of us. Moreover, although early studies of sexual fluidity suggested that it was more common in women than men, recent studies have begun to challenge this view. Two recent studies found no difference in the prevalence of sexual fluidity between men and women, suggesting men and women are similarly fluid. Finally, although most studies have focused on sexual fluidity among adolescents and young adults, there is also evidence fluidity can occur later in life, into middle age and beyond. Finally, an important feature of sexual fluidity is that it can occur in one direction (e.g. an individual may experience increased attractions to men overtime) or might go back and forth over time (e.g. an individual may go through periods of sexually preferring men and then periods of preferring women). For some people, developing feelings for a particular man or woman may be the cause of shifts in one's sexual preference. At other times, shifts in attraction to men or women may feel quite unexpected.*

*In summary, there is a growing body of scientific research finding that for a*

*substantial number of people, sexual orientation is not entirely stable across their lives - it can shift in unexpected ways over time.*”

**Fluidity** Manipulation Check (true or false)

1. Sexual orientation appears to change across time.
2. People can NOT experience genuine changes in their sexual attractions to men and women.
3. Men and women experience changes in their sexual orientation to a similar extent.

The stability manipulation read:

“*Traditionally, an individual’s sexual orientation has been thought to be stable and*

*unchanging overtime. Indeed, over the past several decades, researchers have reaffirmed that people do not report significant changes in their relative attraction to men versus women across their lives. This resilience to change has been labelled “sexual stability.”*

*For instance, a woman who experiences exclusive sexual attraction to men in*

*adolescence and early adulthood is almost certainly going to continue being attracted to only men later in life. The same applies to men and women who are same-sex attracted.*

*Importantly, this stability overtime supports the notion that individuals cannot simply choose their sexual orientation, as attractions are outside of one’s conscious control and fixed once they emerge. Although some individuals may appear to have changed their sexual orientation over time, this seems to represent individuals coming to terms with repressed same-sex desires. For instance, most individuals who transition from a heterosexual to a homosexual identity report that they were always attracted to the same-sex, however may have been initially confused about their sexuality, or chose to present themselves as heterosexual (even engaging in opposite-sex sexual and romantic experiences) in order to avoid discrimination and stigma associated with being homosexual. Lisa Diamond’s longitudinal research (where the same people are followed across a number of years) into female sexuality provides the most dramatic demonstration of sexual stability. Diamond found that, over time, almost no women reported shifts in their relative attraction to men versus women, in the gender of their sexual/romantic partners, or in their sexual identity (e.g. a woman describing herself as a lesbian across multiple years). For example, a typical lesbian woman reported herself as 100% attracted to women at all four stages of the study (over 10 years).*

*This has led to some arguing that stability is a general property of human sexuality.*

*Moreover, although early studies suggested women were more stable, recent studies have*

*challenged this view. Two recent studies found no difference in stability of sexual orientation between men and women, suggesting that men and women are similarly stable. Finally, although most studies have been conducted on adolescents and young adults, there is evidence that people in their middle age and beyond report stability in their attractions. In summary, there is a growing body of scientific research finding that, for the overwhelming majority people, sexual orientation is entirely stable across their lives.*”

**Stability** Manipulation Check (true or false)

1. Sexual orientation appears to remain the same across time
2. People CAN experience genuine changes in their sexual attractions to men and women.
3. Men and women are both similarly stable in their sexual orientation.

Finally the control condition, matched for length, but not content read:

“*A tree is a tall plant with a trunk and branches made of wood. Trees can live for many*

*years. The oldest tree ever discovered is approximately 5,000 years old. The four main parts*

*of a tree are the roots, the trunk, the branches, and the leaves.*

*The roots of a tree are usually under the ground. One case for which this is not true*

*are the roots of the mangrove tree, which are often underwater. A single tree has many roots.*

*The roots carry food and water from the ground through the trunk and branches to the leaves*

*of the tree. They can also breathe in air. Sometimes, roots are specialized into aerial roots,*

*which can also provide support, as is the case with the banyan tree.*

*The trunk is the main body of the tree. The trunk is covered with bark which protects*

*it from damage. Branches grow from the trunk. They spread out so that the leaves can get*

*more sunlight.*

*The leaves of a tree are green most of the time, but they can come in many colours,*

*shapes, and sizes. The leaves take in sunlight and use water and food from the roots to make*

*the tree grow, and reproduce.*

*Trees and shrubs take in water and carbon dioxide and give out oxygen with sunlight*

*to form sugars. This is the opposite of what animals do in respiration. Plants also do some*

*respiration using oxygen the way animals do. They need oxygen as well as carbon dioxide to*

*live.*

*A tree is a plant form that can be found in many different orders and families of*

*plants. Trees show many growth forms, leaf type and shape, bark traits, and organs.*

*The tree form has changed separately in classes of plants that are not related, in*

*response to similar problems (for the tree). With about 100,000 types of trees, the number of*

*tree types in the whole world might be one fourth of all living plant types. Most tree species*

*grow in tropical parts of the world and many of these areas have not been surveyed yet by*

*botanists (who study plants), making species differences and ranges not well understood.*

*The earliest trees were tree ferns, horsetails, and lycophytes, which grew in forests in*

*the Carboniferous period; tree ferns still survive, but the only surviving horsetails and*

*lycophytes are not of tree form. Later, in the Triassic Period, conifers, ginkgos, and cycads*

*appeared, and subsequently flowering plants in the Cretaceous period. Most species of trees*

*today are flowering plants and conifers.*

*A small group of trees growing together is called a grove or copse, and landscape*

*covered by a dense growth of trees is called a forest. Several biotopes are defined largely by*

*the trees that inhabits (e.g. rainforests). A landscape of trees or spaced across grassland*

*(usually grazed or burned over periodically) is called a savanna. A forest of great age is*

*called old growth forest or ancient woodland. A very young tree is called a sampling.”*

**Manipulation Check** (true or false)

1. The four main parts of a tree are the roots, the trunk, the branches, and the leaves.
2. A tree is NOT a tall plant with a trunk and branches made of wood.
3. Trees can live for many years.

# Results

Note. Planned analysis for Study 2 is all reported in text.

**Data Analyses**

Similar to Study 1, for each dependent variable of interest the regression models took the same form. The dummy coded experimental conditions contrasted with the control condition were entered in the first step. Step 2 added the main effects of political orientation and gender and the interactions between political orientation×gender, interactions between political orientation and each experimental condition (continuous, discrete, fluid, stable), and interactions between gender and experimental conditions (continuous, discrete, fluid, stable). As in Study 1, Binomial Logistic Regression was used to analyse sexual orientation uncertainty and willingness to engage in sexual/romantic interactions with the same-sex. We also included a secondary analysis (in Supplemental Materials only) in which we compared continuous versus discrete conditions, and stable versus fluid conditions directly on all dependent variables of interest. Otherwise, these analyses mirrored those described.

As in Study 1, due to multiple comparisons, a Bonferroni correction was applied to each variable, such that a *p*-value of .0167 was used to determine statistical significance.

Results

*Additional Analysis of Discrete versus Continuous/Stable versus Fluid* **Manipulation**

Using hierarchical linear regression models, we undertook follow-up analysis comparing whether those reading the continuous account of sexual orientation would demonstrate more same-sex attraction than those in the discrete account. We additionally examined whether those in the fluid condition would demonstrate more same-sex attraction than those in the stable condition. In both cases we also examined whether these effects would be moderated by gender or political orientation.

*Continuous versus Discrete*

**Sexual Orientation**

We failed to find support for the continuous condition leading to more non-exclusive heterosexual self-ratings than those in the discrete group, *t*(176) *=* .966, *β = -*.059, *p =*.438, CI [-.77, -.07]. Likewise no interactions were significant (*ps.* > .05) (See Supplementary Table 2).

**Secondary Indices**

For sexual orientation uncertainty, no main effect of the continuous manipulation was observed. The second step (including all interactions) was non-significant. Likewise, no main effect of the continuous manipulation was observed on willingness to have same-sex experiences, and the second step failed to reach significance in this case either (*ps*. > .05) (See Supplementary Table 2).

*Fluid versus Stable*

**Sexual Orientation**

Hierarchical linear regression analysis revealed that those in the fluid condition reported significantly more same-sex attraction than those in the stable condition. No significant interactions were observed (See Supplementary Table 3).

**Secondary Indices**

Binomial logistic regression failed to identify any main effect of the fluid versus stable manipulation on sexual orientation uncertainty or willingness to have same-sex/sexual romantic relationships. Likewise, no interactions were observed for either dependent variables (*ps.* > .05)(See Supplementary Table 3).

**References**

1. Mohr, J. J. & Rochlen, A. Measuring attitudes regarding bisexuality in lesbian, gay male, and heterosexual populations. *Journal of Counseling Psychology* **46**, 353-369 (1999).
2. Morrison, M. A. & Morrison, T. G. Development and validation of a scale measuring modern prejudice toward gay men and lesbian women. *Journal of homosexuality* **43**, 15-37 (2003).
3. Arseneau, J. R., Grzanka, P. R., Miles, J. R. & Fassinger, R. E. Development and initial validation of the Sexual Orientation Beliefs Scale (SOBS). *Journal of Counseling Psychology* **60**, 407-420, doi[:http://dx.doi.org/10.1037/a0032799](http://dx.doi.org/10.1037/a0032799) (2013).
4. Mohr, J. J. & Fassinger, R. Information sheet: Lesbian, Gay, and Bisexual Identity Scale. *Unpublished manuscript* (2003).
5. Hayes, A. F. *Introduction to mediation, moderation, and conditional process analysis: A regression-based approach*. (Guilford publications, 2017).


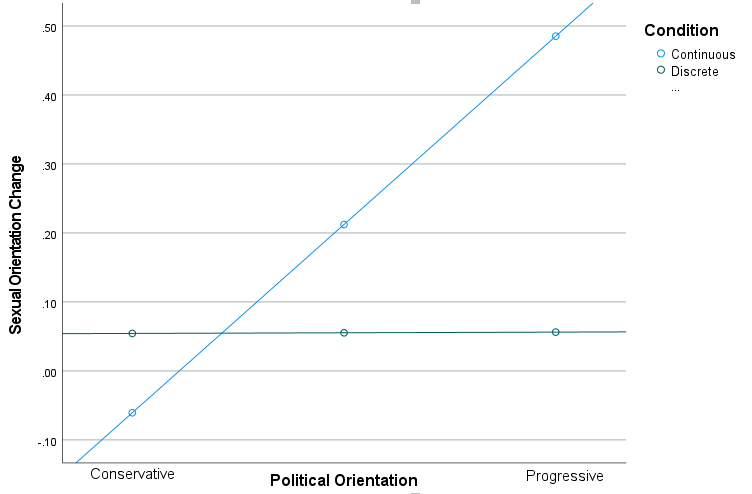


**Supplementary Fig. 1.** Change in sexual orientation self-rating as a function of condition (*control* or *discrete*) and political orientation (*conservative or progressive*). Higher scores indicate a greater shift in sexual orientation from pre- to post-manipulation.

**Supplementary Table 1.** **Study 1.** Effects of reading continuous versus discrete accounts on sexual orientation self-ratings and secondary indices

|  | Sexual Orientation ∆ | | |  | Willingness to have Same Sex Experiences | | | Sexual Orientation Uncertainty | | | |
| --- | --- | --- | --- | --- | --- | --- | --- | --- | --- | --- | --- |
| Variable | β | 95% CI | *sr^2^* |  | β | 95% CI | Exp(β) | | β | 95% CI | Exp(β) |
| Model 1 |  |  |  | Model 1 |  |  |  | |  |  |  |
| *R*^2^ | .008 |  |  | Step χ^2^(2) | 2.368 |  |  | | 3.775 |  |  |
| *F* Change | .933 |  |  | Nagelkerke *R^2^* | .026 |  |  | | .042 |  |  |
| Continuity | .088 | [-.128, .372] | .142 |  | -.565 | [.276, 1.171] | .569 | | .744 | [.994, 4.461] | 2.105 |
| Model 2 |  |  |  | Model 2 |  |  |  | |  |  |  |
| *R*^2^ | .138** |  |  | Step χ^2^(9) | 17.957** |  |  | | 4.46 |  |  |
| *F* Change | 3.700** |  |  | Nagelkerke *R^2^* | .205 |  |  | | .090 |  |  |
| Continuity | -.285 | [-1.249, .467] | .360 |  | .435 | [.099, 24.222] | 1.545 | | .646 | [.112, 32.525] | 1.909 |
| Gender | -.282 | [-1.189, .371] | .301 |  | 2.224 | [.726, 117.758] | 9.246 | | .045 | [.070, 15.598] | 1.046 |
| PO | -1.307 | [-1.215, -.235] | .004** |  | -.619 | [.110, 2.637] | .538 | | -.482 | [.121, 3.166] | .618 |
| Gender x PO | .810 | [.064, .468] | .010* |  | .412 | [.780, 2.921] | 1.509 | | .524 | [.863, 3.304] | 1.688 |
| Continuity x PO | .769 | [.080, .474] | .006** |  | .155 | [.608, 2.240] | 1.167 | | -.129 | [.451, 1.714] | .879 |
| Continuity x Gender | .552 | [-.139, .861] | .155 |  | -.506 | [.119, 3.058] | .603 | | .163 | [.224, 6.181] | 1.177 |
|  |  |  |  |  |  |  |  | |  |  |  |

*Note*. * *p* < .05; ** *p* < .01; *** *p* < .001

PO = political orientation

Continuity: Continuity = 1; Discreteness = 0

**Supplementary Table 2. Study 2.** Effects of reading continuous versus discrete accounts on sexual orientation self-ratings and secondary indices

|  | Sexual Orientation Post-Test | | |  | Willingness to have Same Sex Sexual/Romantic  Experiences | | | Sexual Orientation Uncertainty | | |
| --- | --- | --- | --- | --- | --- | --- | --- | --- | --- | --- |
| Variable | β | 95% CI | *sr^2^* |  | β | 95% CI Exp(β) | | β 95% CI Exp(β) | | |
| Model 1 |  |  |  | Model 1 |  |  |  |  |  |  |
| *R*^2^ | .003 |  |  | Step χ^2^(4) | 3.126 |  |  | 1.816 |  |  |
| *F* Change | .605 |  |  | Nagelkerke *R^2^* | .024 |  |  | .014 |  |  |
| Continuous | -.059 | [ -.419, .182 ] | .194 |  | -.542 | [ .318, 1.064 ] | .582 | -.422 | [ .354, 1.214 ] | .656 |
| Model 2 |  |  |  | Model 2 |  |  |  |  |  |  |
| *R*^2^ | .029 |  |  | Step χ^2^(9) | 17.653 |  |  | 9.595 |  |  |
| *F* Change | .878 |  |  | Nagelkerke *R^2^* | .149 |  |  | .085 |  |  |
| Continuous | -.004 | [-.432, .415 ] | .013 |  | -.840 | [ .169, 1.101 ] | .432 | -.388 | [.270, 1.703 ] | .678 |
| Gender | .199 | [-.561, 1.36] | -.006 |  | -.230 | [ .112, 5.615 ] | .794 | .539 | [.235, 12.477 ] | 1.714 |
| Political Orientation | -.084 | [-.427, .303] | .194 |  | -.449 | [ .290, 1.407] | .638 | -.965 | [.172, .842 ] | .381 |
| Gender x PO | -.108 | [ -.330, .117 ] | .034 |  | -.013 | [ .602, 1.621] | .987 | .080 | [.665, 1.766 ] | 1.083 |
| Continuous x PO | .270 | [-.094, .351 ] | -.002 |  | .596* | [1.077, 3.059] | 1.815 | .697** | [1.223, 3.294 ] | 2.007 |
| Continuous x Gender | -.189 | [-.839, .377 ] | .023 |  | .481 | [.444, 5.890 ] | 1.618 | -.107 | [.249, 3.246 ] | .899 |

|  |  |  |  |  |  |  |  |  |  |  |
| --- | --- | --- | --- | --- | --- | --- | --- | --- | --- | --- |

*Note*. * *p* < .05; ** *p* < .01; *** *p* < .001;

PO = political orientation

Continuity: Continuity = 1; Discreteness = 0

**Supplementary Table 3. Study 2.** Effects of reading fluid versus stable accounts on sexual orientation self-ratings and secondary indices

|  | Sexual Orientation Post-Test | | |  | Willingness to have Same Sex Sexual/Romantic  Experiences | | | Sexual Orientation Uncertainty | | |
| --- | --- | --- | --- | --- | --- | --- | --- | --- | --- | --- |
| Variable | β | 95% CI | *sr^2^* |  | β | 95% CI Exp(β) | | β 95% CI Exp(β) | | |
| Model 1 |  |  |  | Model 1 |  |  |  |  |  |  |
| *R*^2^ | .039*** |  |  | Step χ^2^(4) | .225 |  |  | 1.293 |  |  |
| *F* Change | 7.186 |  |  | Nagelkerke *R^2^* | .0202 |  |  | .010 |  |  |
| Fluid | -.196** | [ -.653,.099] | -.196 |  | -.150 | [ .463, 1.600] | .861 | -.368 | [ .367,1.306] | .692 |
| Model 2 |  |  |  | Model 2 |  |  |  |  |  |  |
| *R*^2^ | .029 |  |  | Step χ^2^(9) | 52.151 |  |  | 5.167 |  |  |
| *F* Change | 1.100 |  |  | Nagelkerke *R^2^* | .170 |  |  | .049 |  |  |
| Fluid | -.141 | [-.671,.131] | -.196 |  | -.155 | [ .262, 2.796] | .856 | .212 | [.480,3.187] | 1.237 |
| Gender | .210 | [-.476,1.280] | .062 |  | 1.902 | [ .709, 63.237] | 6.698 | 1.909 | [ .879,51.815] | 6.747 |
| Political Orientation | -.438 | [ -.633, .035] | -.079 |  | -.009 | [ .414, 2.370] | .991 | .108 | [ .498,2.494] | 1.114 |
| Gender x PO | .202 | [ -.017, .385] | .020 |  | .043 | [ .611, 1.784] | 1.044 | .234 | [ .783, 2.039] | 1.264 |
| Fluid x PO | .248 | [ -.091, .314] | -.056 |  | .184 | [ .720, 2.006] | 1.202 | -.116 | [ .549,1.445] | .891 |
| Fluid x Gender | -.176 | [ -.765, .354] | -.016 |  | -.203 | [ .192, 3.463] | .816 | -1.113 | [ .088,1.227] | .328 |

|  |  |  |  |  |  |  |  |  |  |  |
| --- | --- | --- | --- | --- | --- | --- | --- | --- | --- | --- |

*Note*. * *p* < .05; ** *p* < .01; *** *p* < .001;

PO = political orientation

Fluid: Fluidity = 1; Stable = 0
